# Supplementary material for: Allosteric regulation and crystallographic fragment screening of SARS-CoV-2 NSP15 endoribonuclease
Source: Nucleic Acids Res. 2023 Apr 28;51(10):5255–70. doi: 10.1093/nar/gkad314 (PMC10250223; doi:10.1093/nar/gkad314)
Supplement: gkad314_Supplemental_Files [file gkad314_supplemental_files.zip › Supplementary datasets.zip!/231/Refine_8/01-BUSTER/Cycle-4/LIST.html]

Refining

**BUSTER 2.X**: A suite of programs for macromolecular structure refinement by maximum-likelihood and Bayesian methods  
Copyright © 1992-2020 Global Phasing Ltd.  
All rights reserved.  
Authors: G. Bricogne, E. Blanc, M. Brandl, C. Flensburg, P. Keller, W. Paciorek, P. Roversi, O. Smart, C. Vonrhein, T. Womack  

Please reference use of the software as:

- BUSTER-TNT 2.X, Global Phasing Ltd, Sheraton House, Cambridge CB3 0AX, UK

Version: 2.10.3 for linux64-ifort made on May 20 2020 at 01:14:34  
Licenced to: Diamond Light Source, Scientific Software  

```
---


### Parsing of the BUSTER cardfile

TITLE : autoBUSTER refinement

   Cell information

   Symmetry information

 Using CCP4 X-ray form factors.

   Missing structure chemical composition

 Using ATOM block for chemical composition.


   Shell information

### Parsing of the TNT cardfile


---


### Reading observations file

 Number of reflexions in input:  85622

      Number of accepted reflexions:  81660

      Number of rejected reflexions:      0

   Reflexions in test set:            4077

   Reflexions in working set:        77583


---


### 1. Generating initial model

 2.10 * Shannon sampling rate. NX NY NZ:   360  360  240


  Effective Shannon rates are:    2.50    2.50    2.26


   Allocated BUFFER of size:    119.312 Mb.

   Initial Geometry Information


 No missing atoms declared


   Summary of model volumes

 No missing atoms declared: no prior computed


 Computing bulk solvent model scattering


 NCSMASK PDB radius for solvent mask:     2.15 Angstrom

 running PDBSET (setting cell and spacegroup P1) ... done
 running NCSMASK [1] (creating mask, SMOOTH 0.921083) ... done
 running MAPDUMP (to check if reduction to unit cell is needed) ... done
 running MAPMASK (converting mask to map) ... done

   Generation of babslv by theta-filter

   Electron density in frg mask:  0.420 Å-1

 FOBS  put on rough absolute scale by Wilson plot :
           K        =  0.37723E+02
           B        =    -11.881

 

### Cycle 0001: MaxLik scaling and refinement


   MaxLik Scaling Cycle 0001

   Wilson plot

   Statistics in resolution bins just after scaling
```

|  |  |
| --- | --- |
|  | - Rxpct factors plot- Rxpct factors table- <Log-Lik Gain>- Ln Sigma A- Correlation Coefficients |

```
   <Log-Likelihood> of null-hypothesis

Structure Factor Amplitudes


   Average Figure of Merit

 Switching off refinement of overall temperature factor


 Added B =     0.4405 Å2 to all atoms in fragment


 Added B =     0.4405 Å2 to B_SOLV

```
gelly refinement module
 Authors (2004-2009) O. S. Smart, C. Flensburg, W. Paciorek, C. Vonrhein, 
                     M. Brandl, T. O. Womack and G. Bricogne
         (2010-2011) O. S. Smart, C. Flensburg, W. Paciorek, C. Vonrhein, 
                     T. O. Womack and G. Bricogne
         (2012-2014) O. S. Smart, C. Flensburg, W. Paciorek, C. Vonrhein, 
                     and G. Bricogne
         (2015-2020) G. Bricogne, C. Flensburg, W. Paciorek, C. Vonrhein
 Copyright © 2004-2020 Global Phasing Ltd. 
 All rights reserved.
 gelly module compilation date May 20 2020 01:11:20
 
 gelly uses routines from TNT geometry. References: 
     Tronrud, D. E., Ten Eyck, L. F., Matthews, B. W. (1987)
     "An Efficient General-Purpose Least-Squares Refinement Program 
     for Macromolecular Structures". Acta Crystallogr A, 43:489-501.
     Tronrud, D. E. (1997). "The TNT Refinement Package", 
     in Macromolecular Crystallography, Part B, Eds Charlie Carter 
     and Robert Sweet, Methods in Enzymology, 277:306-319.
 wildcard matching function by Julian Robichaux http://www.nsftools.com

 No -random_seed flag found. Random number stream initialised using:
2147483562
2147483398

 Control flags set to:  -autoncs -hydrogens_input_occ2zero \
                     -hydrogens_output_occ2heavy -screen \
                     100 -screen_sigma \
                     3.0 -keeppdb \
                     1000000 -taomonitor_verbose \
                     2 
 

 ############### Starting setup via TNT routines

 Setting up tnt routines including license check:

                   TNT Stereochemistry Program
 Version: 5.22.3.0
 Licence for Diamond Light Source, Scientific Software is valid
 
 Using TNT geometry routines to load the tnt control file: gelly.tnt
 Using TNT routines to load initial coordinates from file fragment.cor
 Expecting 6138 atoms

 Found     2195 "NOTE BUSTER_RESIDUE_ATOM_DATA" cards
 Have copied information for    6138 atoms from TNT to gelly's store 
 
 The TNT WEIGHT cards used by this run:
 -----WEIGHT       BOND     2.0
 -----WEIGHT       ANGLE    2.0
 -----WEIGHT       IMPROPER 0.0
 -----WEIGHT       TORSION  2.0
 -----WEIGHT       PSEUDO   0.0
 -----WEIGHT       TRIGONAL 2.0
 -----WEIGHT       PLANE    5.0
 -----WEIGHT       CONTACT  5.0
 -----WEIGHT       BCORREL  10.0
 -----WEIGHT       CHIRAL   5.0
 -----WEIGHT       NCS      50.0
 -----WEIGHT       IDEAL    4.0
 -----WEIGHT       RFACTOR  5.24
 
 Dictionary versions picked up from NOTE BUSTER_RESTRAINT_DICT cards as: 
 ------ protgeo_eh99.dat (V1.6.4.17) 20191001  STANDARD AMINO ACID DICTIONARY. BONDS AND ANGLES FROM ENGH AND HUBER EH99. OTHER VALUES BASED ON PREVIOUS TNT OR TAKEN FROM CCP4. INCLUDES HYDROGEN ATOMS.
 ------ exoticaa.dat (V1.3.4.15) 20200327  COLLECTION OF NON-STANDARD AMINO ACIDS, MAINLY EH91 WITHOUT IDEAL DISTANCE INFO
 ------ nuclgeo.dat (V1.13.4.8) 20190909
 ------ bcorrel.dat (V1.15.10.2) 20190616
 ------ contact.dat (V1.15.12.10) 20191121
 ------ idealdist_contact.dat (V1.3.4.12) 20191001  IDEAL-DISTANCE CONTACT TERM DATA AS USED IN PROLSQ. VALUES USED HERE ARE BASED ON DATA HARVESTING OF PDB AS OF 20190109.
 ------ restraints for LIG (Unknown) from cif dictionary POB0120.cif
 ------ restraints for CIT (CITRIC ACID) from cif dictionary CIT.cif; generated by GRADE_PDB_LIGAND 1.2.12 (October 4 2016) using MOGUL 1.7.2(RC1), CSD as538be, with quantum mechanics RM1 ((DatabaseID PDB-CIT))
 ------ assume.dat (V1.9.4.2) 20180705
 
 Have picked up spacegroup name for pdb output as 'P 63      '
 Setting up Chain/Residue arrays
 
 Have command line specification -autoncs*
     Have found       1 NCS relations
     Pairwise matching residue number and type between chains: 
                    A        B        S    
          A         .      100%       -   
          B                  .        -   
          S                           .   
       Table key--                                                       
          all   : two chains have 100% match and same number of residues 
         multi  : ditto and they contribute to a multi chain ncs cluster 
          ***%  : two chains have >80% matching residue number/type      
           -    : chains appear to be unrelated                          
      chain A     have identified ncs to     1 other chains. So will weight each autoncs by (       6.666/#)=      6.6660
      chain B     have identified ncs to     1 other chains. So will weight each autoncs by (       6.666/#)=      6.6660
      chain S     have identified ncs to     0 other chains. 
     For .Gelly cards produced see below - after pruning
 
 Setting up BUSTER_SET groups
 Have found    89 BUSTER_SET cards
 Now silently applying automatic/dictionary set definitions. For a verbose listing use '-verbose_set' flag.
 Applying user defined set definitions:
 =====NOTE BUSTER_SET ncsautoXcld = Water --> set 'ncsautoXcld' now has   581 atoms 
 =====NOTE BUSTER_SET ncsautoABset = Chain_A \ ncsautoXcld --> set 'ncsautoABset' now has  2785 atoms 
 Have defined    48 sets: 
      set:                 'All' has   6138 atoms in   1282 residues - automatically defined
      set:               'Empty' has      0 atoms in      0 residues - automatically defined
      set:            'ExoticAA' has      0 atoms in      0 residues - defined by dictionary file
      set:           'ExoticNUC' has      0 atoms in      0 residues - defined by dictionary file
      set:              'NotHET' has   5514 atoms in    698 residues - defined by dictionary file
      set:             'Chain_A' has   2785 atoms in    351 residues - automatically defined
      set:             'Chain_B' has   2772 atoms in    350 residues - automatically defined
      set:             'Chain_S' has    581 atoms in    581 residues - automatically defined
      set:               'Water' has    581 atoms in    581 residues - defined by dictionary file
      set:          'StdProtein' has   5514 atoms in    698 residues - defined by dictionary file
      set:             'Protein' has   5514 atoms in    698 residues - defined by dictionary file
      set:                'Back' has   2794 atoms in    698 residues - defined by dictionary file
      set:                'Side' has   2720 atoms in    650 residues - defined by dictionary file
      set:        'RnaCanonical' has      0 atoms in      0 residues - defined by dictionary file
      set:        'DnaCanonical' has      0 atoms in      0 residues - defined by dictionary file
      set:     'RnaDnaCanonical' has      0 atoms in      0 residues - defined by dictionary file
      set:     'DnaRnaCanonical' has      0 atoms in      0 residues - defined by dictionary file
      set:         'RnaExtended' has      0 atoms in      0 residues - defined by dictionary file
      set:         'DnaExtended' has      0 atoms in      0 residues - defined by dictionary file
      set:      'RnaDnaExtended' has      0 atoms in      0 residues - defined by dictionary file
      set:      'DnaRnaExtended' has      0 atoms in      0 residues - defined by dictionary file
      set:                 'Dna' has      0 atoms in      0 residues - defined by dictionary file
      set:                 'Rna' has      0 atoms in      0 residues - defined by dictionary file
      set:              'RnaDna' has      0 atoms in      0 residues - defined by dictionary file
      set:              'DnaRna' has      0 atoms in      0 residues - defined by dictionary file
      set:             'Nucleic' has      0 atoms in      0 residues - defined by dictionary file
      set:             'Polymer' has   5514 atoms in    698 residues - defined by dictionary file
      set: 'BufferWeichenberger' has     13 atoms in      1 residues - defined by dictionary file
      set:    'BufferAdditional' has      0 atoms in      0 residues - defined by dictionary file
      set:              'Buffer' has     13 atoms in      1 residues - defined by dictionary file
      set:         'MetalAlkali' has      0 atoms in      0 residues - defined by dictionary file
      set:  'MetalAlkalineEarth' has      0 atoms in      0 residues - defined by dictionary file
      set:     'MetalTransition' has      0 atoms in      0 residues - defined by dictionary file
      set: 'MetalPostTransition' has      0 atoms in      0 residues - defined by dictionary file
      set:    'MetalLanthanides' has      0 atoms in      0 residues - defined by dictionary file
      set:               'Metal' has      0 atoms in      0 residues - defined by dictionary file
      set:         'SugarAlphaD' has      0 atoms in      0 residues - defined by dictionary file
      set:         'SugarAlphaL' has      0 atoms in      0 residues - defined by dictionary file
      set:               'Sugar' has      0 atoms in      0 residues - defined by dictionary file
      set:               'Other' has     43 atoms in      3 residues - defined by dictionary file
      set:            'Hydrogen' has      0 atoms in      0 residues - defined by dictionary file
      set:         'nonHydrogen' has   6138 atoms in   1282 residues - defined by dictionary file
      set:             'OccZero' has      0 atoms in      0 residues - defined by dictionary file
      set:          'OccNonZero' has   6138 atoms in   1282 residues - defined by dictionary file
      set:             'OccFull' has   6076 atoms in   1278 residues - defined by dictionary file
      set:          'OccNonFull' has     62 atoms in      4 residues - defined by dictionary file
      set:         'ncsautoXcld' has    581 atoms in    581 residues - user specified
      set:        'ncsautoABset' has   2785 atoms in    351 residues - user specified
 No command line arguments '-occ_allhet' or '-occ_restyp' so will not setup automated occcupancy refinement
 
 Interpreting CONSTANT and FREE cards: 
 =====CONSTANT     OCC
 Have found and interpreted      1 CONSTANT cards in the TNT input. 
 Before any COMBINE - Number of atoms with fixed XYZ =     0
 Before any COMBINE - Number of atoms with fixed B   =     0
 Before any COMBINE - Number of atoms with fixed OCC =  6138
 Before any COMBINE - Number of atoms with fixed BIJ =   581
                            cf total number of atoms =  6138
 
 There are no COMBINE cards to be found in the TNT card input 
 
 There are no NOTE BUSTER_COMBINE_RES cards to be found in the TNT card input 

 ############### Working out full connectivity list


 ############### Working out equivalent atoms


 Residue with most atoms given below:
        A|501:O01(LIG)      
        A|501:C02(LIG)      
        A|501:N03(LIG)      
        A|501:C04(LIG)      
        A|501:C05(LIG)      
        A|501:C06(LIG)      
        A|501:C07(LIG)      
        A|501:C08(LIG)      
        A|501:C09(LIG)      
        A|501:C10(LIG)      
        A|501:C11(LIG)      
        A|501:N12(LIG)      
        A|501:C13(LIG)      
        A|501:C14(LIG)      
        A|501:C15(LIG)      


 Have no '-equiv_check_plus' argument - so will take atom type into
 account when checking for equivalence of otherwise identical atoms.
 

 ############### Looking at dihedrals


 ############### Looking at chirals

 NOTE BUSTER_CHIRAL_ANGLECUT card found - read value=    -31.00 degs from it
 NOTE BUSTER_CHIRAL_SIGMA card found - read value=      1.70 degs from it
 RANGE allowed for B variables in refinement:   from      3.00000 to    300.00000

 ############### Preparing for PDB output


 ############### Processing extra restraints explicitly defined

 No 'NOTE BUSTER_TARGET' cards or -target?? args found (used to read coordinates files for soft ncs to external)
 No 'NOTE BUSTER_OCCSUM'     cards found - so not using sum of occupancy restraints
 No 'NOTE BUSTER_DISTANCE'   cards found - so not setting up any additional atom-atom distance restraints
 No 'NOTE BUSTER_UTILANGLE'  cards found - so not setting up any additional restraints on any angle
 No 'NOTE BUSTER_UTILTOR'    cards found - so not setting up any additional sinusoidal restraints on torsions
 
 --- Have some similarity definitions 
     Similarity restraints on RMSD use superposition routines from: 
     Coutsias, E.A., Seok, C., Dill, K.A.(2004)
     "Using quaternions to calculate RMSD", J. Comput. Chem., 25:1849-1857 

 Have found    1 'NOTE BUSTER_SIM_DEFINE' cards 
     Maximum number of chains involved in any SIM group (excluding the template)=     1


 ########## Interpreting card: NOTE BUSTER_SIM_DEFINE ncsautoAB ncsautoABset B
     Number of atoms in template set=  2785
     Number of related chains (other than template) in SIM set=    1
     List of chains to be restrained (excluding template)=   B   
     Pairwise RMSD, XYZ in Angstroms, for SIM group: ncsautoAB
                 A         B   
     A         -----     ----- 
     B         3.138     ----- 
     Overall RMSD,XYZ for the SIM group is     3.138 Angstroms
     Pairwise rms deviation of [individual B's minus their set/chain average] in Angs**2
                 A         B         Bavg
     A         -----     -----     78.78
     B         22.98     -----     60.81
     Overall RMSD,B   for the SIM group is    22.982 Angs**2
     The number of common atoms for the above fits:
                 A         B   
     A         -----     ----- 
     B          2772     ----- 
     (compared to the template set that has    2785 atoms in it)
     List of atoms that are absent in one or more chains for this group: 
                                    A    B   
                    C1      401     +    -
                    O1      401     +    -
                    O2      401     +    -
                    C2      401     +    -
                    C3      401     +    -
                    O7      401     +    -
                    C4      401     +    -
                    C5      401     +    -
                    O3      401     +    -
                    O4      401     +    -
                    C6      401     +    -
                    O5      401     +    -
                    O6      401     +    -
     (key  +  atom present for this chain 
           -  atom absent  for this chain)
 transforms for each of the superpositions orthogonal matrix r11 r12 r13 r21 r22 r23 r31 r32 r33 tx ty tz (PDBSET syntax): 
TRANS_AB -0.878234 -0.477857 -0.018927 -0.477989  0.878361  0.002906  0.015235  0.011599 -0.999817    34.289    32.781    15.462
 reverse transforms 
TRANS_BA -0.878234 -0.477989  0.015235 -0.477857  0.878361  0.011599 -0.018927  0.002906 -0.999817    45.548   -12.588    16.013
 
 Setting up LSSR interatomic distance restraints
 Interpreting card: NOTE BUSTER_SIM_RESTRAIN_LSSR ncsautoAB 6.666
        setting up interatomic distance pair restraints with:
        Dmin=         0.00
        Dmax=         5.50
        sigma=        0.20
        Vmax=         3.00
        weight=       6.67
        number of distance pairs defined for this set is      34479
 Have not supplied either '-sim_swap_equiv' or '-sim_swap_equiv_plus' argument 
   so will not try swaping equivalent atoms to improve NCS and/or target agreement.
 
 NCS by LSSR outlier analysis
 -autoncs option automatic NCS prune routine -------- 
 Residues pruned out by gradient condition:
          A|135     A|136     A|147     A|148     A|149     A|150     A|151
          A|158     A|159     A|160     A|166     A|167     A|168     A|169
          A|170     A|171     A|172     A|173     A|174     A|175     A|182
          A|187     A|188     A|189     A|212     A|215     A|216     A|217
          A|219     A|220     A|253     A|259     A|260     A|264     A|344
          A|345     A|501     B|135     B|136     B|145     B|147     B|148
          B|149     B|150     B|151     B|158     B|159     B|160     B|166
          B|167     B|168     B|169     B|170     B|171     B|172     B|173
          B|174     B|175     B|182     B|187     B|188     B|189     B|212
          B|215     B|216     B|217     B|219     B|220     B|253     B|259
          B|260     B|264     B|344     B|345     B|501
  
 Residues pruned out by function/max condition:
          A|149!    A|168!    A|169!    A|170!    A|171!    A|172!    A|173!
          A|187!    A|188!    A|264!    A|345!   A|ENDA     A|501!    B|149!
          B|168!    B|169!    B|170!    B|171!    B|172!    B|173!    B|187!
          B|188!    B|264!    B|345!   B|ENDA     B|501!
  
 Total number of residues for which LSSR restraints have been pruned out is       77 out of      700 participating
 Number of LSSR restraints pruned out is      4749 this is   13.8% of the total           34479
  
 -autoncs .Gelly cards suitable for adaptation for manual definition/pruning
           (note auto pruning is done in a slightly different way so exact results may differ slightly)
 -autoncs option .Gelly cards also written to file  auto_gelly_cards.txt
NOTE BUSTER_SET ncsautoXcld = Water    # card added - produced by -autoncs
NOTE BUSTER_SET ncsautoXcld = ncsautoXcld + { A|135 A|136 A|147 A|148 A|149 }    # card added - produced by -autoncs
NOTE BUSTER_SET ncsautoXcld = ncsautoXcld + { A|150 A|151 A|158 A|159 A|160 }    # card added - produced by -autoncs
NOTE BUSTER_SET ncsautoXcld = ncsautoXcld + { A|166 A|167 A|168 A|169 A|170 }    # card added - produced by -autoncs
NOTE BUSTER_SET ncsautoXcld = ncsautoXcld + { A|171 A|172 A|173 A|174 A|175 }    # card added - produced by -autoncs
NOTE BUSTER_SET ncsautoXcld = ncsautoXcld + { A|182 A|187 A|188 A|189 A|212 }    # card added - produced by -autoncs
NOTE BUSTER_SET ncsautoXcld = ncsautoXcld + { A|215 A|216 A|217 A|219 A|220 }    # card added - produced by -autoncs
NOTE BUSTER_SET ncsautoXcld = ncsautoXcld + { A|253 A|259 A|260 A|264 A|344 }    # card added - produced by -autoncs
NOTE BUSTER_SET ncsautoXcld = ncsautoXcld + { A|345 A|ENDA A|501 }    # card added - produced by -autoncs
NOTE BUSTER_SET ncsautoABset = Chain_A \ ncsautoXcld    # card added - produced by -autoncs
NOTE BUSTER_SIM_DEFINE ncsautoAB ncsautoABset B    # card added - produced by -autoncs
NOTE BUSTER_SIM_RESTRAIN_LSSR ncsautoAB 6.666    # card added - produced by -autoncs

 LSSR function contribution before pruning     41270.2
                             after pruning     10340.6
 Graph of which residues pruned from LSSR: ncs_pruned.mtv
 Graph of LSSR function outliers ncs_function_outliers.mtv
 Graph of LSSR gradient outliers ncs_gradient_outliers.mtv
 LSSR - analysis of distance difference distribution for NCS
         DeltaD less than 0.5 Angs:  proportion of population= 98.57% average DeltaD of subset     0.008 Angs
 Histogram of LSSR deltaD distribution ncs_histogram.mtv
 
 Interpreting card: NOTE BUSTER_TLS_SET tls1 { A|* }
 Interpreting card: NOTE BUSTER_TLS_SET tls2 { B|* }
 Interpreting card: NOTE BUSTER_TLS_SET tls1 { A|* }
 Interpreting card: NOTE BUSTER_TLS_T   tls1     -1.42739E-01 -2.31034E-02  1.80298E-01 -3.76226E-02  2.26092E-02  1.74795E-02
 Interpreting card: NOTE BUSTER_TLS_L   tls1      1.45453E+00  1.61782E+00  8.45083E-01  9.10505E-02 -6.05438E-01  5.77972E-01
 Interpreting card: NOTE BUSTER_TLS_S   tls1      2.23750E-01 -2.44115E-01  3.38595E-01 -6.35630E-02  3.30303E-03 -2.70940E-01 -8.61559E-02 -8.56052E-02
 Interpreting card: NOTE BUSTER_TLS_O   tls1     -2.33111E+01 -8.37601E+01  1.13266E+01
 Interpreting card: NOTE BUSTER_TLS_SET tls2 { B|* }
 Interpreting card: NOTE BUSTER_TLS_T   tls2     -1.96623E-01 -1.35074E-01  3.50236E-01  1.18743E-02  4.75488E-02 -1.48386E-02
 Interpreting card: NOTE BUSTER_TLS_L   tls2      1.48927E+00  9.09257E-01  1.09932E+00  7.78817E-01 -9.29773E-01 -7.15920E-01
 Interpreting card: NOTE BUSTER_TLS_S   tls2     -3.97061E-02  1.25383E-01 -1.13212E-01  1.50247E-01  1.99842E-01  3.65144E-02 -6.77258E-02  2.74050E-02
 Interpreting card: NOTE BUSTER_TLS_O   tls2      2.21121E+01 -7.20987E+01  3.46915E+00
 Interpreting card: NOTE BUSTER_TLS_FIX tls1 ALL
 Interpreting card: NOTE BUSTER_TLS_FIX tls2 ALL


 TLS information. Total number of groups:     2


 Group   Natoms    FixRB   FixTLS   Group Tag    Group Spec
     1     2785      Y        Y     tls1    { A|* }
     2     2772      Y        Y     tls2    { B|* }
 
NOTE BUSTER_TLS_SET tls1 { A|* }
NOTE BUSTER_TLS_T   tls1     -1.42739E-01 -2.31034E-02  1.80298E-01 -3.76226E-02  2.26092E-02  1.74795E-02
NOTE BUSTER_TLS_L   tls1      1.45453E+00  1.61782E+00  8.45083E-01  9.10505E-02 -6.05438E-01  5.77972E-01
NOTE BUSTER_TLS_S   tls1      2.23750E-01 -2.44115E-01  3.38595E-01 -6.35630E-02  3.30303E-03 -2.70940E-01 -8.61559E-02 -8.56052E-02
NOTE BUSTER_TLS_O   tls1     -2.33111E+01 -8.37601E+01  1.13266E+01
NOTE BUSTER_TLS_FIX tls1 ALL
 
NOTE BUSTER_TLS_SET tls2 { B|* }
NOTE BUSTER_TLS_T   tls2     -1.96623E-01 -1.35074E-01  3.50236E-01  1.18743E-02  4.75488E-02 -1.48386E-02
NOTE BUSTER_TLS_L   tls2      1.48927E+00  9.09257E-01  1.09932E+00  7.78817E-01 -9.29773E-01 -7.15920E-01
NOTE BUSTER_TLS_S   tls2     -3.97061E-02  1.25383E-01 -1.13212E-01  1.50247E-01  1.99842E-01  3.65144E-02 -6.77258E-02  2.74050E-02
NOTE BUSTER_TLS_O   tls2      2.21121E+01 -7.20987E+01  3.46915E+00
NOTE BUSTER_TLS_FIX tls2 ALL


 TLS: mmCIF group specification begin


#
loop_
_pdbx_refine_tls_group.id
_pdbx_refine_tls_group.refine_tls_id
_pdbx_refine_tls_group.beg_PDB_ins_code
_pdbx_refine_tls_group.beg_auth_asym_id
_pdbx_refine_tls_group.beg_auth_seq_id
_pdbx_refine_tls_group.end_PDB_ins_code
_pdbx_refine_tls_group.end_auth_asym_id
_pdbx_refine_tls_group.end_auth_seq_id
_pdbx_refine_tls_group.selection
_pdbx_refine_tls_group.selection_details
   1   1 . A   -1 . A  346 . '{ A|* }'
   2   1 . A  401 . A  501 . '{ A|* }'
   3   2 . B   -1 . B  346 . '{ B|* }'
   4   2 . B  501 . B  501 . '{ B|* }'
#


 TLS: mmCIF group specification end


 Interpreting card: NOTE BUSTER_ANISO_SET Empty
 Interpreting card: NOTE BUSTER_ANISO_WEIGHT SCA  16.0
 Interpreting card: NOTE BUSTER_ANISO_WEIGHT SPH   0.6    0.6
 Interpreting card: NOTE BUSTER_ANISO_WEIGHT SIM   7.8    4.8    2.6    0
 Interpreting card: NOTE BUSTER_ANISO_WEIGHT RIG  21.5   21.5    3.7    0


 After TLS and ANISO: Number of atoms with fixed/combined xyz =     0
 After TLS and ANISO: Number of atoms with fixed/combined B   =     0
 After TLS and ANISO: Number of atoms with fixed/combined OCC =  6138
 After TLS and ANISO: Number of atoms with fixed/combined BIJ =  6138
 After TLS and ANISO: Number of atoms in a TLS group          =  5557
                                     cf total number of atoms =  6138
 

 ############### Setup state vector

 No free_vect_ulim_xyz argument found: using default of  100.000
 No free_vect_llim_xyz argument found: using default of -100.000
 Number of parameters added for TLS description                 =       0
 Number of parameters to describe molecular conformation        =   24552

 ############### Setup free vector

 Number of free parameters to be adjusted in refinement   =   24552

 ############### Setting up own geometry


 No command line argument '-tnt_fn' so will use gelly to do geometry function calculations

 ============= Call TNT functions


 ============= Get TNT symmetry information

 
      Getting symmetry operators from TNT. 
          gelly will classify symmetry using pdb-like convention: 
               SYMOP   SYMMETRY
              NNNMMM   OPERATOR
                1555   X,Y,Z
                2555   -Y,X-Y,Z
                3555   -X+Y,-X,Z
                4555   -X,-Y,1/2+Z
                5555   Y,-X+Y,1/2+Z
                6555   X-Y,X,1/2+Z
          where NNN -> operator number and MMM -> translation vector
 

 ============= Own Gelly geometry/restraints setup


 ----------- Identify bond-types


    Setting up GEOMBONDREC_TYPE_HASH* to be able to map a given bond type (e.g.
    "single") to an internal integer (GBOND_TYPE=1) etc.

    Using default  GEOMBONDREC_TYPE_HASH_STR='SING* 1 DOUB* 2 TRIP* 3 AROM* 4 DELO* 4 METAL* 5':
    to override default use a NOTE BUSTER_GEOMBONDREC_TYPE_HASH_STR gelly card.

 ----------- Classify torsion into peptide omega and other


 ----------- Setup of non-bonded terms

 Ideal-distance contact term setup:
    Restraint function constants (normally these values are from NOTE BUSTER_IDEAL_CONTACT_* cards)
    Sigma for general contacts      0.200 Angs
    Sigma for 1-4 contacts          0.100 Angs (contacts across a torsion)
    Sigma for 1-5 contacts          0.150 Angs
    Sigma for 1-N contacts          0.200 Angs
    Sigma for HBOND pairs           0.100 Angs (contacts between HB_types A and D or B's)
    Contact distance increment for each atom in HBOND          -0.150 Angs 
    Contact distance increment for H-bond acceptor              0.100 Angs 
    Contact distance increment for 1-4 with an O atom          -0.050 Angs 
    Contact distance increment for 1-4 with an N atom          -0.180 Angs 
    Contact distance increment for 1-4 with an C atom          -0.370 Angs 
    Contact distance increment for 1-4 with an S atom          -0.150 Angs 
    Contact distance increment for 1-4 with an P atom          -0.150 Angs 
    Contact distance increment for 1-4 with any other atom     -0.150 Angs 
    All atoms matched for CCP4 atom type.
    Number of CCP4 atom types used is         23 (H-bound and not H-bound counted separately)

 Setting up short/ideal-distance contacts
   Using gelly special position handling. (For TNT instead rerun with argument '-special_tnt')

   No command line argument '-special_all' specified. We will auto-generate
   EXCLUDE cards only for atom-atom contacts across symmetry (i.e. on special
   position) when this involves a contact within a residue to itself.

   Have set up non-bonded exclusions - 1-3 contacts
   Atom A|27:CA         makes the most 1-3 contacts:  6

   Have set up non-bonded exclusions - 1-4 contacts
   Atom A|27:N          makes the most 1-4 contacts:  7

   Have set up non-bonded exclusions - 1-5 contacts
   Atom A|28:C.A        makes the most 1-5 contacts: 10

 ############################################################################################################
 ###################################################### setting up contact terms
 ############################################################################################################


 ============= Call Gelly versions of TNT functions

 WARNING: 1-4 contact without 1-(2,3)-4 data for A|401:O2(CIT)        and A|401:C3(CIT)        X-X
 WARNING: 1-4 contact without 1-(2,3)-4 data for A|401:C2(CIT)        and A|401:O6(CIT)        X-X
 WARNING: 1-4 contact without 1-(2,3)-4 data for A|401:O7(CIT)        and A|401:O5(CIT)        X-X
 WARNING: 1-4 contact without 1-(2,3)-4 data for A|401:C4(CIT)        and A|401:O6(CIT)        X-X
 WARNING: 1-4 contact without 1-(2,3)-4 data for A|501:O01(LIG)       and A|501:C15(LIG)       X-X
 WARNING: 1-4 contact without 1-(2,3)-4 data for A|501:C02(LIG)       and A|501:C09(LIG)       X-X
 WARNING: 1-4 contact without 1-(2,3)-4 data for A|501:C04(LIG)       and A|501:C11(LIG)       X-X
 WARNING: 1-4 contact without 1-(2,3)-4 data for B|501:C04(LIG)       and B|501:C11(LIG)       X-X

       TERM      weight      number      TNT_fn    Gelly_fn        diff
       BOND       2.000        5676     997.888     997.888       0.000
       ANGL       2.000        7707    5676.717    5676.717       0.000
       IMPR       0.000           0       0.000       0.000       0.000
       TORS       2.000        1979    2875.359    2875.359      -0.000
       TRIG       2.000           0       0.000       0.000       0.000
       PLAN       5.000         966    2263.994    2263.994       0.000
       CONT       5.000           0    2183.770       0.000    2183.770
       BCOR      10.000        5630    2462.594    4496.414   -2033.819


 ============= Call Gelly geometry functions

   Switching torsion functional form to sinusoidal rather than split harmonic functional.
      if you want this not to happen then rerun with '-torsharm'
      Weight for sinusoidal torsion=     2.000
   BCOR function/gradient will be split between H and non-H atoms (as for any atom pair). Use '-bcorrel_all_in_hydrogens' to change this.

 ----------- Fix B-correlation restraints

 
 Checking for bonds that do not have a bcorrel restraint defined
 We will wipe all existing BCORrels if OCC=0.0 atom(s) is/are involved. This can be' switched off via the '-bcorrel_nowipe_zeroocc' flag.
 We will wipe all existing BCORrels if only non-hydrogen atom(s) with fixed B(iso) are involved. This can be switched off via the '-bcorrel_nowipe_fixb_nonhydrogens' flag.
 We will keep all existing BCORrels if hydrogen atom(s) with fixed B(iso) is/are involved. This can be switched off via the '-bcorrel_wipe_fixb_hydrogens' flag.
 No -bcorrel_missing_sigma found. So will set sigma to     2.5 Ang**2 for any bond with no existing BCORrel
 No -bcorrel_missing_sigma_hydrogens found. So will set sigma to     1.0 Ang**2 for any bond with no existing BCORrel involving hydrogens

 No active BCORels found involving OCC=0.0 atom(s)


 No active BCORels found involving fixed-B (only) non-hydrogen atom(s)

    add a default BCOR for bond A|401:C1(CIT) A|401:C2(CIT)
    add a default BCOR for bond A|401:C1(CIT) A|401:O1(CIT)
    add a default BCOR for bond A|401:C1(CIT) A|401:O2(CIT)
    add a default BCOR for bond A|401:C2(CIT) A|401:C3(CIT)
    add a default BCOR for bond A|401:C3(CIT) A|401:C4(CIT)
    add a default BCOR for bond A|401:C3(CIT) A|401:C6(CIT)
    add a default BCOR for bond A|401:C3(CIT) A|401:O7(CIT)
    add a default BCOR for bond A|401:C4(CIT) A|401:C5(CIT)
    add a default BCOR for bond A|401:C5(CIT) A|401:O3(CIT)
    add a default BCOR for bond A|401:C5(CIT) A|401:O4(CIT)
    add a default BCOR for bond A|401:C6(CIT) A|401:O5(CIT)
    add a default BCOR for bond A|401:C6(CIT) A|401:O6(CIT)
    add a default BCOR for bond A|501:O01(LIG) A|501:C02(LIG)
    add a default BCOR for bond A|501:C02(LIG) A|501:N03(LIG)
    add a default BCOR for bond A|501:C02(LIG) A|501:C11(LIG)
    add a default BCOR for bond A|501:N03(LIG) A|501:C04(LIG)
    add a default BCOR for bond A|501:C04(LIG) A|501:C05(LIG)
    add a default BCOR for bond A|501:C04(LIG) A|501:C09(LIG)
    add a default BCOR for bond A|501:C05(LIG) A|501:C06(LIG)
    add a default BCOR for bond A|501:C05(LIG) A|501:C10(LIG)
    add a default BCOR for bond A|501:C06(LIG) A|501:C07(LIG)
    add a default BCOR for bond A|501:C07(LIG) A|501:C08(LIG)
    add a default BCOR for bond A|501:C08(LIG) A|501:C09(LIG)
    add a default BCOR for bond A|501:C10(LIG) A|501:C11(LIG)
    add a default BCOR for bond A|501:C11(LIG) A|501:N12(LIG)
    add a default BCOR for bond A|501:C11(LIG) A|501:C15(LIG)
    add a default BCOR for bond A|501:N12(LIG) A|501:C13(LIG)
    add a default BCOR for bond A|501:C13(LIG) A|501:C14(LIG)
    add a default BCOR for bond A|501:C14(LIG) A|501:C15(LIG)
    add a default BCOR for bond B|501:O01(LIG) B|501:C02(LIG)
    add a default BCOR for bond B|501:C02(LIG) B|501:N03(LIG)
    add a default BCOR for bond B|501:C02(LIG) B|501:C11(LIG)
    add a default BCOR for bond B|501:N03(LIG) B|501:C04(LIG)
    add a default BCOR for bond B|501:C04(LIG) B|501:C05(LIG)
    add a default BCOR for bond B|501:C04(LIG) B|501:C09(LIG)
    add a default BCOR for bond B|501:C05(LIG) B|501:C06(LIG)
    add a default BCOR for bond B|501:C05(LIG) B|501:C10(LIG)
    add a default BCOR for bond B|501:C06(LIG) B|501:C07(LIG)
    add a default BCOR for bond B|501:C07(LIG) B|501:C08(LIG)
    add a default BCOR for bond B|501:C08(LIG) B|501:C09(LIG)
    add a default BCOR for bond B|501:C10(LIG) B|501:C11(LIG)
    add a default BCOR for bond B|501:C11(LIG) B|501:N12(LIG)
    add a default BCOR for bond B|501:C11(LIG) B|501:C15(LIG)
    add a default BCOR for bond B|501:N12(LIG) B|501:C13(LIG)
    add a default BCOR for bond B|501:C13(LIG) B|501:C14(LIG)
    add a default BCOR for bond B|501:C14(LIG) B|501:C15(LIG)

 Have added a total of        46 BCORrels for bonds which do not already have a BCORrel


 No bonds (for potential BCORrel addition) were skipped due to both atoms having constant/fixed B(iso)


 ----------- Deactivate hydrogen-free planes if hydrogen atoms present

 A total of       0 planes have been deactivated because they are duplicated hydrogen-free and with-hydrogen forms.

 ----------- Handle peptide omega angle(s)

      Torsion angles split into peptide omega and other
         Number of peptide omega angles is       698
         Number of other torsion is             1281
      Peptide omega angles are monitored rather than directly restrained (PLANE used instead).

 ----------- Fix cis-PRO

 Now apply the fix to get correct restraints for cis PRO residues
   Have got      11 bonds records and      26 angles
   from GEOMETRY cards for the special PROC residue
   Weight for ideal-distance contact term=     4.000

 ----------- Prepare SSBOND/LINK for output PDB file


 ----------- Prepare for QM


 ----------- Prepare symmetry in utility distance restraints


 ############### Setting up Rhofit contacts (if needed)

 No ligand-(fixed protein) contact term will be used

 ############### Working out initial function and gradient


   MaxLik Scaling Cycle 0002
 
 Initial functional value = 5.041472E+05 rms gradient=      60.9872
 X-ray contribution to function value = 4.693673E+05
 Geometry contribution (inc ncs)      =      34779.9
 Geometry function summary
     term                           number     weight       rms        GooF      function     fn/numb
     ================================================================================================
     BOND bond lengths (angs)       5676       2.000      0.0059       0.296       997.89       0.176
     ANGL bond angles (degs)        7707       2.000        1.02       0.607      5676.72       0.737
     TORS split-harmonic tors       1281       0.000       16.89       1.059         0.00       0.000
     SINTOR sinusoidal tors         1281       2.000       16.89                  2202.55       1.719
     PLAN general plane (angs)       966       5.000      0.0137       0.685      2263.99       2.344
     BCORrelations (angs^2)         5676      10.000       0.790       0.282      4513.83       0.795
     CONT (bad contacts)               0       5.000                                 0.00
     IDEAL(ideal-dist contact)      5028       4.000                              8750.20
     CHIRAL (gelly semiharmon)       740       5.000                                34.10
     SIM similarity restraint for NCS and/or TARGET                              10340.64
     OCCSUM restraint on sum of occupanies                                           0.00       as none defined
     DISTAN utility restraints interatomic distance                                  0.00       as none defined
     UTILANGLE utility angle restraints                                              0.00       as none defined
     UTILTOR utility restraints on torsion angles                                    0.00       as none defined
     Peptide omega torsion angles                           2.61 degs - monitor only
 
 WARNING: Have |delta/sigma| deviations > 5.0 sigma. Number of outliers for each term: 
 WARNING:         2 bond lengths. Worst is     6.7 sigs     1.25 Angs A|501:C05=C04 (LIG)
 WARNING:         5 bond angles.  Worst is    21.9 sigs    54.78 degs A|501:C09=C04=C05 (LIG)
 WARNING:         1 planes.       Worst is     9.4 sigs     0.19 Angs A|501:N03=C04=C05=C06=C07=C08=C09=C10 (LIG)
 WARNING:         1 idealD contacts. Worst     8.3 sigs     2.01 Angs A|501:C09=C11 (LIG)

 Weighted rms      0.0059         1.020  (temporary rms bond and angle for BUSTER to pick up!)
 Weighted intra-residual rms(bond) for StdProtein (excluding H)      0.0051
 Weighted intra-residual rms(bond) for Buffer     (excluding H)      0.0037

 ############### Running initial check


 Initial Geometry Sanity Check

 Check for duplicate bonds: no duplicates found
 
 Check for duplicate or missing bond angles: all are OK
 Check for bond between an atom and itself not involving symmetry:  all are OK
 *** WARNING: There are 1 bad contacts where Delta/sigma is greater than      6.5
 *** WARNING: Looking at each one in turn to see whether it is likely to indicate a setup problem with geometry restraints.

        1 dist=   2.01 idealD=   3.67 sigma=   0.20 abs(Delta/sigma)=    8.29
          Between atoms   A|501:C09 (LIG)         and   A|501:C11 (LIG)         (C-C)
          occupancies=     0.44        0.44
 [check-033]
          This is a contact within a residue - so probably just a distorted
          residue?
 GEOMETRY CHECK OVERALL:  WARN


 ############### Creating geometry/screen report


 ############### Option "tao" - run Tao optimizer

 TAO: Toolkit for Advanced Optimization. Reference: 
     Munson, T., Sarich, J., Wild, S., Benson, S., McInnes, L.C. (2018)
     "TAO User Manual (Revision 3.9)", Mathematics and Computer Science
     Division, Argonne National Laboratory
     ANL/MCS-TM-322, http://www.mcs.anl.gov/petsc/
 
 Have found -keeppdb flag so will write out a .pdb file every 1000000 its during opt
 Will apply TAO optimization 
 Maximum number of iterations      100
 Convergence test on Grms       4.0000 (-glim)
 Limit on rms displacement to original position (rmsD2init): NONE test inactive (-dlim)

    analysing argument no 0 : -autoncs with j=0
    analysing argument no 1 : -hydrogens_input_occ2zero with j=0
    analysing argument no 2 : -hydrogens_output_occ2heavy with j=0
    analysing argument no 3 : -screen with j=0
    analysing argument no 4 : 100 with j=0
    analysing argument no 5 : -screen_sigma with j=0
    analysing argument no 6 : 3.0 with j=0
    analysing argument no 7 : -keeppdb with j=0
    analysing argument no 8 : 1000000 with j=0
    analysing argument no 9 : -taomonitor_verbose with j=0
    found argument starting with "-tao" : -taomonitor_verbose
    analysing argument no 10 : 2 with j=1
    found value after -tao/-options_/-viewer_ argument : "2"
 PETSC/TAO: setting option "-taomonitor_verbose" = "2"


   MaxLik Scaling Cycle 0003
   Iters     Tot_Funct    Xray_Funct    Geom_Funct          Grms     rmsD2init     maxD2init             R         Rfree           LLG       LLGfree     rms(bond)    rms(angle)       Opttag_H
   Iters             K   B_IMPF_FRAG        K_MISS   B_TEMP_MISS   K_IMPF_MISS   B_IMPF_MISS        K_SOLV   B_TEMP_SOLV   K_IMPF_SOLV   B_IMPF_SOLV        B_TEMP     B_TEMP_11     B_TEMP_12     B_TEMP_22     B_TEMP_13     B_TEMP_23     B_TEMP_33      K_WILSON      B_WILSON      FGCYCLES       Scatag_H
   Iters          BOND         ANGLE      IMPROPER       TORSION    SINTORSION      TRIGONAL        PLANAR  BCORRELATION       CONTACT     IDEALDIST        CHIRAL    SIMILARITY       OCCSSUM  UTILDISTANCE     UTILANGLE   UTILTORSION        RHOFIT        ADP-01        ADP-02        ADP-03        ADP-04        ADP-05        ADP-06        ADP-07        ADP-08        ADP-09        ADP-10      RHOFITPC            QM       Funtag_H
       0  5.041472E+05  4.693673E+05       34779.9       60.9872  0.000000E+00  0.000000E+00      0.224727      0.242683  0.000000E+00  0.000000E+00    0.00594928       1.01986       Opttag_D
       0       45.2107       2.78213       1.00000  0.000000E+00       1.00000  0.000000E+00      0.807301       63.9981       1.00000       23.9017  0.000000E+00       1.39177  0.000000E+00       1.39177  0.000000E+00  0.000000E+00      -2.78353       45.1306       1.58302        2       Scatag_D
       0       997.888       5676.72  0.000000E+00  0.000000E+00       2202.55  0.000000E+00       2263.99       4513.83  0.000000E+00       8750.20       34.0953       10340.6 -1.000000E+10 -1.000000E+10 -1.000000E+10 -1.000000E+10 -1.000000E+10  0.000000E+00  0.000000E+00  0.000000E+00  0.000000E+00  0.000000E+00  0.000000E+00  0.000000E+00  0.000000E+00  0.000000E+00  0.000000E+00  0.000000E+00  0.000000E+00       Funtag_D
   Geometry Restraint Deviations SCREEN Information

 

 An atom has moved more than     1.50 A from the initial/last
 position - if this was an accepted trial point, probably need to update the
 non-bonded contact list.


   MaxLik Scaling Cycle 0004

   MaxLik Scaling Cycle 0005

   MaxLik Scaling Cycle 0006

   Wilson plot

   Statistics in resolution bins after refinement cycle 0001
```


|  |  |
| --- | --- |
|  | - Rxpct factors plot- Rxpct factors table- <Log-Lik Gain>- Ln Sigma A- Correlation Coefficients |


```
   Iters     Tot_Funct    Xray_Funct    Geom_Funct          Grms     rmsD2init     maxD2init             R         Rfree           LLG       LLGfree     rms(bond)    rms(angle)
   Iters             K   B_IMPF_FRAG        K_MISS   B_TEMP_MISS   K_IMPF_MISS   B_IMPF_MISS        K_SOLV   B_TEMP_SOLV   K_IMPF_SOLV   B_IMPF_SOLV        B_TEMP     B_TEMP_11     B_TEMP_12     B_TEMP_22     B_TEMP_13     B_TEMP_23     B_TEMP_33      K_WILSON      B_WILSON      FGCYCLES
   Iters          BOND         ANGLE      IMPROPER       TORSION    SINTORSION      TRIGONAL        PLANAR  BCORRELATION       CONTACT     IDEALDIST        CHIRAL    SIMILARITY       OCCSSUM  UTILDISTANCE     UTILANGLE   UTILTORSION        RHOFIT        ADP-01        ADP-02        ADP-03        ADP-04        ADP-05        ADP-06        ADP-07        ADP-08        ADP-09        ADP-10      RHOFITPC            QM
       1  5.033668E+05  4.685000E+05       34866.8       55.6636  8.784553E-04    0.00898033      0.224268      0.242551    0.00213370  5.752305E-04    0.00611182       1.02115       Opttag_D
       1       45.1806       2.73890       1.00000  0.000000E+00       1.00000  0.000000E+00      0.807065       63.7592       1.00000       24.0238  0.000000E+00       1.38081  0.000000E+00       1.38081  0.000000E+00  0.000000E+00      -2.76161       45.1306       1.58302        3       Scatag_D
       1       1053.16       5691.12  0.000000E+00  0.000000E+00       2201.75  0.000000E+00       2288.08       4496.78  0.000000E+00       8754.40       34.9496       10346.6 -1.000000E+10 -1.000000E+10 -1.000000E+10 -1.000000E+10 -1.000000E+10  0.000000E+00  0.000000E+00  0.000000E+00  0.000000E+00  0.000000E+00  0.000000E+00  0.000000E+00  0.000000E+00  0.000000E+00  0.000000E+00  0.000000E+00  0.000000E+00       Funtag_D
   Geometry Restraint Deviations SCREEN Information

 

   MaxLik Scaling Cycle 0007

   Wilson plot

   Statistics in resolution bins after refinement cycle 0002
```


|  |  |
| --- | --- |
|  | - Rxpct factors plot- Rxpct factors table- <Log-Lik Gain>- Ln Sigma A- Correlation Coefficients |


```
   Iters     Tot_Funct    Xray_Funct    Geom_Funct          Grms     rmsD2init     maxD2init             R         Rfree           LLG       LLGfree     rms(bond)    rms(angle)
   Iters             K   B_IMPF_FRAG        K_MISS   B_TEMP_MISS   K_IMPF_MISS   B_IMPF_MISS        K_SOLV   B_TEMP_SOLV   K_IMPF_SOLV   B_IMPF_SOLV        B_TEMP     B_TEMP_11     B_TEMP_12     B_TEMP_22     B_TEMP_13     B_TEMP_23     B_TEMP_33      K_WILSON      B_WILSON      FGCYCLES
   Iters          BOND         ANGLE      IMPROPER       TORSION    SINTORSION      TRIGONAL        PLANAR  BCORRELATION       CONTACT     IDEALDIST        CHIRAL    SIMILARITY       OCCSSUM  UTILDISTANCE     UTILANGLE   UTILTORSION        RHOFIT        ADP-01        ADP-02        ADP-03        ADP-04        ADP-05        ADP-06        ADP-07        ADP-08        ADP-09        ADP-10      RHOFITPC            QM
       2  5.027630E+05  4.677489E+05       35014.1       40.7834    0.00182016     0.0131506      0.223823      0.242410    0.00398112    0.00114675    0.00632012       1.02155       Opttag_D
       2       45.1521       2.70268       1.00000  0.000000E+00       1.00000  0.000000E+00      0.806852       63.6133       1.00000       24.0843  0.000000E+00       1.37115  0.000000E+00       1.37115  0.000000E+00  0.000000E+00      -2.74229       45.1306       1.58302        1       Scatag_D
       2       1126.17       5695.59  0.000000E+00  0.000000E+00       2200.83  0.000000E+00       2320.72       4509.56  0.000000E+00       8763.81       35.5984       10361.8 -1.000000E+10 -1.000000E+10 -1.000000E+10 -1.000000E+10 -1.000000E+10  0.000000E+00  0.000000E+00  0.000000E+00  0.000000E+00  0.000000E+00  0.000000E+00  0.000000E+00  0.000000E+00  0.000000E+00  0.000000E+00  0.000000E+00  0.000000E+00       Funtag_D
   Geometry Restraint Deviations SCREEN Information

 
       3  5.021943E+05  4.665153E+05       35679.0       45.4847    0.00363718     0.0275892      0.223070      0.242147    0.00701557    0.00207780    0.00674121       1.02709       Opttag_D
       3       45.1037       2.64481       1.00000  0.000000E+00       1.00000  0.000000E+00      0.806511       63.4143       1.00000       24.1550  0.000000E+00       1.35672  0.000000E+00       1.35672  0.000000E+00  0.000000E+00      -2.71344       45.1306       1.58302        1       Scatag_D
       3       1281.23       5757.54  0.000000E+00  0.000000E+00       2199.14  0.000000E+00       2389.26       4815.28  0.000000E+00       8790.31       36.3482       10409.9 -1.000000E+10 -1.000000E+10 -1.000000E+10 -1.000000E+10 -1.000000E+10  0.000000E+00  0.000000E+00  0.000000E+00  0.000000E+00  0.000000E+00  0.000000E+00  0.000000E+00  0.000000E+00  0.000000E+00  0.000000E+00  0.000000E+00  0.000000E+00       Funtag_D
       4  5.019231E+05  4.651664E+05       36756.7       72.9789    0.00587829     0.0474338      0.222226      0.241820     0.0103337    0.00308454    0.00723224       1.04384       Opttag_D
       4       45.0500       2.58363       1.00000  0.000000E+00       1.00000  0.000000E+00      0.806157       63.2351       1.00000       24.2079  0.000000E+00       1.34163  0.000000E+00       1.34163  0.000000E+00  0.000000E+00      -2.68326       45.1306       1.58302        1       Scatag_D
       4       1474.68       5946.86  0.000000E+00  0.000000E+00       2197.00  0.000000E+00       2482.85       5297.75  0.000000E+00       8831.09       36.7921       10489.7 -1.000000E+10 -1.000000E+10 -1.000000E+10 -1.000000E+10 -1.000000E+10  0.000000E+00  0.000000E+00  0.000000E+00  0.000000E+00  0.000000E+00  0.000000E+00  0.000000E+00  0.000000E+00  0.000000E+00  0.000000E+00  0.000000E+00  0.000000E+00       Funtag_D
       5  5.015561E+05  4.650223E+05       36533.8       38.3538    0.00621714     0.0516185      0.222128      0.241754     0.0106882    0.00323976    0.00717465       1.03179       Opttag_D
       5       45.0427       2.57731       1.00000  0.000000E+00       1.00000  0.000000E+00      0.806117       63.2324       1.00000       24.2034  0.000000E+00       1.34111  0.000000E+00       1.34111  0.000000E+00  0.000000E+00      -2.68222       45.1306       1.58302        1       Scatag_D
       5       1451.29       5810.26  0.000000E+00  0.000000E+00       2196.58  0.000000E+00       2498.26       5206.74  0.000000E+00       8840.34       36.5101       10493.8 -1.000000E+10 -1.000000E+10 -1.000000E+10 -1.000000E+10 -1.000000E+10  0.000000E+00  0.000000E+00  0.000000E+00  0.000000E+00  0.000000E+00  0.000000E+00  0.000000E+00  0.000000E+00  0.000000E+00  0.000000E+00  0.000000E+00  0.000000E+00       Funtag_D
       6  5.012024E+05  4.645532E+05       36649.2       36.4779    0.00732820     0.0638090      0.221811      0.241568     0.0118421    0.00369582    0.00720673       1.03447       Opttag_D
       6       45.0212       2.55690       1.00000  0.000000E+00       1.00000  0.000000E+00      0.805990       63.2123       1.00000       24.1978  0.000000E+00       1.33783  0.000000E+00       1.33783  0.000000E+00  0.000000E+00      -2.67566       45.1306       1.58302        1       Scatag_D
       6       1464.30       5840.57  0.000000E+00  0.000000E+00       2195.41  0.000000E+00       2545.54       5182.99  0.000000E+00       8867.40       36.1509       10516.9 -1.000000E+10 -1.000000E+10 -1.000000E+10 -1.000000E+10 -1.000000E+10  0.000000E+00  0.000000E+00  0.000000E+00  0.000000E+00  0.000000E+00  0.000000E+00  0.000000E+00  0.000000E+00  0.000000E+00  0.000000E+00  0.000000E+00  0.000000E+00       Funtag_D
       7  5.008662E+05  4.638399E+05       37026.3       41.5221    0.00904586     0.0821892      0.221336      0.241297     0.0135965    0.00437980    0.00732132       1.04022       Opttag_D
       7       44.9918       2.52645       1.00000  0.000000E+00       1.00000  0.000000E+00      0.805798       63.1788       1.00000       24.1947  0.000000E+00       1.33114  0.000000E+00       1.33114  0.000000E+00  0.000000E+00      -2.66229       45.1306       1.58302        1       Scatag_D
       7       1511.23       5905.69  0.000000E+00  0.000000E+00       2193.76  0.000000E+00       2617.77       5294.20  0.000000E+00       8910.02       36.3145       10557.3 -1.000000E+10 -1.000000E+10 -1.000000E+10 -1.000000E+10 -1.000000E+10  0.000000E+00  0.000000E+00  0.000000E+00  0.000000E+00  0.000000E+00  0.000000E+00  0.000000E+00  0.000000E+00  0.000000E+00  0.000000E+00  0.000000E+00  0.000000E+00       Funtag_D
       8  5.005174E+05  4.622310E+05       38286.4       88.3325     0.0134097      0.129190      0.220268      0.240649     0.0175543    0.00599543    0.00766132       1.07336       Opttag_D
       8       44.9329       2.46008       1.00000  0.000000E+00       1.00000  0.000000E+00      0.805358       63.1269       1.00000       24.1914  0.000000E+00       1.31313  0.000000E+00       1.31313  0.000000E+00  0.000000E+00      -2.62626       45.1306       1.58302        1       Scatag_D
       8       1654.85       6287.87  0.000000E+00  0.000000E+00       2189.76  0.000000E+00       2795.93       5638.53  0.000000E+00       9028.89       38.4661       10652.1 -1.000000E+10 -1.000000E+10 -1.000000E+10 -1.000000E+10 -1.000000E+10  0.000000E+00  0.000000E+00  0.000000E+00  0.000000E+00  0.000000E+00  0.000000E+00  0.000000E+00  0.000000E+00  0.000000E+00  0.000000E+00  0.000000E+00  0.000000E+00       Funtag_D
       9  5.000233E+05  4.614479E+05       38575.4       35.6377     0.0156774      0.153355      0.219748      0.240313     0.0194805    0.00676899    0.00774688       1.05659       Opttag_D
       9       44.9070       2.42882       1.00000  0.000000E+00       1.00000  0.000000E+00      0.805139       63.1118       1.00000       24.1901  0.000000E+00       1.30303  0.000000E+00       1.30303  0.000000E+00  0.000000E+00      -2.60606       45.1306       1.58302        1       Scatag_D
       9       1692.02       6092.99  0.000000E+00  0.000000E+00       2188.03  0.000000E+00       2891.02       5870.26  0.000000E+00       9095.39       39.9563       10705.7 -1.000000E+10 -1.000000E+10 -1.000000E+10 -1.000000E+10 -1.000000E+10  0.000000E+00  0.000000E+00  0.000000E+00  0.000000E+00  0.000000E+00  0.000000E+00  0.000000E+00  0.000000E+00  0.000000E+00  0.000000E+00  0.000000E+00  0.000000E+00       Funtag_D
      10  4.997981E+05  4.612627E+05       38535.4       28.0529     0.0163013      0.160340      0.219636      0.240204     0.0199360    0.00706601    0.00773591       1.05631       Opttag_D
      10       44.9034       2.42140       1.00000  0.000000E+00       1.00000  0.000000E+00      0.805052       63.1049       1.00000       24.2048  0.000000E+00       1.29954  0.000000E+00       1.29954  0.000000E+00  0.000000E+00      -2.59908       45.1306       1.58302        1       Scatag_D
      10       1687.23       6089.76  0.000000E+00  0.000000E+00       2187.73  0.000000E+00       2905.31       5813.73  0.000000E+00       9114.63       40.6898       10696.4 -1.000000E+10 -1.000000E+10 -1.000000E+10 -1.000000E+10 -1.000000E+10  0.000000E+00  0.000000E+00  0.000000E+00  0.000000E+00  0.000000E+00  0.000000E+00  0.000000E+00  0.000000E+00  0.000000E+00  0.000000E+00  0.000000E+00  0.000000E+00       Funtag_D
      11  4.994474E+05  4.606360E+05       38811.4       34.4810     0.0185861      0.184339      0.219246      0.239841     0.0214777    0.00797442    0.00777364       1.06126       Opttag_D
      11       44.8893       2.39675       1.00000  0.000000E+00       1.00000  0.000000E+00      0.804777       63.0997       1.00000       24.2356  0.000000E+00       1.28874  0.000000E+00       1.28874  0.000000E+00  0.000000E+00      -2.57748       45.1306       1.58302        1       Scatag_D
      11       1703.73       6146.91  0.000000E+00  0.000000E+00       2186.44  0.000000E+00       2967.63       5882.39  0.000000E+00       9188.02       42.7553       10693.5 -1.000000E+10 -1.000000E+10 -1.000000E+10 -1.000000E+10 -1.000000E+10  0.000000E+00  0.000000E+00  0.000000E+00  0.000000E+00  0.000000E+00  0.000000E+00  0.000000E+00  0.000000E+00  0.000000E+00  0.000000E+00  0.000000E+00  0.000000E+00       Funtag_D
      12  4.992116E+05  4.597310E+05       39480.5       70.9479     0.0224644      0.221704      0.218664      0.239293     0.0237036    0.00927004    0.00788207       1.08192       Opttag_D
      12       44.8680       2.36204       1.00000  0.000000E+00       1.00000  0.000000E+00      0.804334       63.1309       1.00000       24.2641  0.000000E+00       1.27388  0.000000E+00       1.27388  0.000000E+00  0.000000E+00      -2.54777       45.1306       1.58302        1       Scatag_D
      12       1751.59       6388.62  0.000000E+00  0.000000E+00       2184.56  0.000000E+00       3071.94       6013.32  0.000000E+00       9316.26       44.7921       10709.5 -1.000000E+10 -1.000000E+10 -1.000000E+10 -1.000000E+10 -1.000000E+10  0.000000E+00  0.000000E+00  0.000000E+00  0.000000E+00  0.000000E+00  0.000000E+00  0.000000E+00  0.000000E+00  0.000000E+00  0.000000E+00  0.000000E+00  0.000000E+00       Funtag_D
      13  4.988719E+05  4.593863E+05       39485.6       33.2411     0.0241688      0.237051      0.218436      0.239070     0.0245517    0.00979605    0.00783918       1.07146       Opttag_D
      13       44.8607       2.34918       1.00000  0.000000E+00       1.00000  0.000000E+00      0.804144       63.1579       1.00000       24.2708  0.000000E+00       1.26839  0.000000E+00       1.26839  0.000000E+00  0.000000E+00      -2.53677       45.1306       1.58302        1       Scatag_D
      13       1732.58       6265.67  0.000000E+00  0.000000E+00       2183.74  0.000000E+00       3114.44       6050.19  0.000000E+00       9376.39       45.5167       10717.1 -1.000000E+10 -1.000000E+10 -1.000000E+10 -1.000000E+10 -1.000000E+10  0.000000E+00  0.000000E+00  0.000000E+00  0.000000E+00  0.000000E+00  0.000000E+00  0.000000E+00  0.000000E+00  0.000000E+00  0.000000E+00  0.000000E+00  0.000000E+00       Funtag_D
      14  4.986360E+05  4.590526E+05       39583.4       25.9080     0.0260425      0.252091      0.218204      0.238824     0.0253724     0.0103186    0.00781243       1.07385       Opttag_D
      14       44.8538       2.33701       1.00000  0.000000E+00       1.00000  0.000000E+00      0.803926       63.2022       1.00000       24.2704  0.000000E+00       1.26389  0.000000E+00       1.26389  0.000000E+00  0.000000E+00      -2.52778       45.1306       1.58302        1       Scatag_D
      14       1720.78       6293.64  0.000000E+00  0.000000E+00       2182.88  0.000000E+00       3154.52       6018.11  0.000000E+00       9443.76       45.7799       10723.9 -1.000000E+10 -1.000000E+10 -1.000000E+10 -1.000000E+10 -1.000000E+10  0.000000E+00  0.000000E+00  0.000000E+00  0.000000E+00  0.000000E+00  0.000000E+00  0.000000E+00  0.000000E+00  0.000000E+00  0.000000E+00  0.000000E+00  0.000000E+00       Funtag_D
      15  4.984234E+05  4.586479E+05       39775.4       30.4283     0.0284408      0.269738      0.217910      0.238519     0.0263678     0.0109125    0.00779800       1.07883       Opttag_D
      15       44.8480       2.32278       1.00000  0.000000E+00       1.00000  0.000000E+00      0.803649       63.2652       1.00000       24.2676  0.000000E+00       1.25861  0.000000E+00       1.25861  0.000000E+00  0.000000E+00      -2.51721       45.1306       1.58302        1       Scatag_D
      15       1714.43       6352.17  0.000000E+00  0.000000E+00       2181.94  0.000000E+00       3199.87       6015.05  0.000000E+00       9530.67       46.4948       10734.8 -1.000000E+10 -1.000000E+10 -1.000000E+10 -1.000000E+10 -1.000000E+10  0.000000E+00  0.000000E+00  0.000000E+00  0.000000E+00  0.000000E+00  0.000000E+00  0.000000E+00  0.000000E+00  0.000000E+00  0.000000E+00  0.000000E+00  0.000000E+00       Funtag_D
      16  4.982095E+05  4.578516E+05       40357.9       64.3611     0.0332540      0.300056      0.217326      0.237943     0.0283267     0.0119177    0.00784373       1.09766       Opttag_D
      16       44.8466       2.29631       1.00000  0.000000E+00       1.00000  0.000000E+00      0.803098       63.3944       1.00000       24.2666  0.000000E+00       1.24782  0.000000E+00       1.24782  0.000000E+00  0.000000E+00      -2.49565       45.1306       1.58302        1       Scatag_D
      16       1734.59       6575.85  0.000000E+00  0.000000E+00       2180.05  0.000000E+00       3272.23       6075.86  0.000000E+00       9708.83       48.6684       10761.8 -1.000000E+10 -1.000000E+10 -1.000000E+10 -1.000000E+10 -1.000000E+10  0.000000E+00  0.000000E+00  0.000000E+00  0.000000E+00  0.000000E+00  0.000000E+00  0.000000E+00  0.000000E+00  0.000000E+00  0.000000E+00  0.000000E+00  0.000000E+00       Funtag_D
      17  4.979047E+05  4.575210E+05       40383.7       28.4156     0.0353145      0.310535      0.217077      0.237711     0.0291399     0.0122781    0.00781817       1.08872       Opttag_D
      17       44.8512       2.28614       1.00000  0.000000E+00       1.00000  0.000000E+00      0.802861       63.4557       1.00000       24.2662  0.000000E+00       1.24328  0.000000E+00       1.24328  0.000000E+00  0.000000E+00      -2.48655       45.1306       1.58302        1       Scatag_D
      17       1723.31       6469.16  0.000000E+00  0.000000E+00       2179.47  0.000000E+00       3293.97       6111.08  0.000000E+00       9784.96       49.6454       10772.1 -1.000000E+10 -1.000000E+10 -1.000000E+10 -1.000000E+10 -1.000000E+10  0.000000E+00  0.000000E+00  0.000000E+00  0.000000E+00  0.000000E+00  0.000000E+00  0.000000E+00  0.000000E+00  0.000000E+00  0.000000E+00  0.000000E+00  0.000000E+00       Funtag_D
      18  4.977072E+05  4.572769E+05       40430.3       23.5617     0.0365773      0.313237      0.216891      0.237558     0.0297404     0.0124983    0.00782877       1.08924       Opttag_D
      18       44.8607       2.27937       1.00000  0.000000E+00       1.00000  0.000000E+00      0.802696       63.4920       1.00000       24.2710  0.000000E+00       1.23950  0.000000E+00       1.23950  0.000000E+00  0.000000E+00      -2.47900       45.1306       1.58302        1       Scatag_D
      18       1727.98       6475.40  0.000000E+00  0.000000E+00       2178.93  0.000000E+00       3290.94       6105.12  0.000000E+00       9828.72       50.3217       10772.9 -1.000000E+10 -1.000000E+10 -1.000000E+10 -1.000000E+10 -1.000000E+10  0.000000E+00  0.000000E+00  0.000000E+00  0.000000E+00  0.000000E+00  0.000000E+00  0.000000E+00  0.000000E+00  0.000000E+00  0.000000E+00  0.000000E+00  0.000000E+00       Funtag_D
      19  4.974823E+05  4.568280E+05       40654.4       32.5692     0.0390822      0.318850      0.216541      0.237263     0.0308446     0.0129144    0.00788040       1.09345       Opttag_D
      19       44.8818       2.26756       1.00000  0.000000E+00       1.00000  0.000000E+00      0.802387       63.5704       1.00000       24.2783  0.000000E+00       1.23129  0.000000E+00       1.23129  0.000000E+00  0.000000E+00      -2.46259       45.1306       1.58302        1       Scatag_D
      19       1750.85       6525.49  0.000000E+00  0.000000E+00       2177.79  0.000000E+00       3290.39       6160.77  0.000000E+00       9916.63       51.5928       10780.8 -1.000000E+10 -1.000000E+10 -1.000000E+10 -1.000000E+10 -1.000000E+10  0.000000E+00  0.000000E+00  0.000000E+00  0.000000E+00  0.000000E+00  0.000000E+00  0.000000E+00  0.000000E+00  0.000000E+00  0.000000E+00  0.000000E+00  0.000000E+00       Funtag_D
      20  4.973025E+05  4.561956E+05       41106.9       51.3336     0.0432083      0.328372      0.216029      0.236816     0.0324003     0.0135712    0.00797905       1.10344       Opttag_D
      20       44.9214       2.25274       1.00000  0.000000E+00       1.00000  0.000000E+00      0.801924       63.7215       1.00000       24.2808  0.000000E+00       1.21782  0.000000E+00       1.21782  0.000000E+00  0.000000E+00      -2.43563       45.1306       1.58302        1       Scatag_D
      20       1794.96       6645.24  0.000000E+00  0.000000E+00       2176.21  0.000000E+00       3298.82       6271.76  0.000000E+00       10061.9       53.0314       10805.0 -1.000000E+10 -1.000000E+10 -1.000000E+10 -1.000000E+10 -1.000000E+10  0.000000E+00  0.000000E+00  0.000000E+00  0.000000E+00  0.000000E+00  0.000000E+00  0.000000E+00  0.000000E+00  0.000000E+00  0.000000E+00  0.000000E+00  0.000000E+00       Funtag_D
      21  4.970801E+05  4.560156E+05       41064.5       27.2572     0.0445452      0.331170      0.215873      0.236658     0.0328431     0.0137915    0.00796031       1.09822       Opttag_D
      21       44.9383       2.24948       1.00000  0.000000E+00       1.00000  0.000000E+00      0.801775       63.7830       1.00000       24.2783  0.000000E+00       1.21280  0.000000E+00       1.21280  0.000000E+00  0.000000E+00      -2.42560       45.1306       1.58302        1       Scatag_D
      21       1786.54       6582.54  0.000000E+00  0.000000E+00       2175.58  0.000000E+00       3301.94       6249.25  0.000000E+00       10106.6       53.1286       10809.0 -1.000000E+10 -1.000000E+10 -1.000000E+10 -1.000000E+10 -1.000000E+10  0.000000E+00  0.000000E+00  0.000000E+00  0.000000E+00  0.000000E+00  0.000000E+00  0.000000E+00  0.000000E+00  0.000000E+00  0.000000E+00  0.000000E+00  0.000000E+00       Funtag_D
      22  4.968771E+05  4.557683E+05       41108.8       23.3836     0.0466219      0.336401      0.215647      0.236409     0.0334512     0.0141345    0.00794222       1.09971       Opttag_D
      22       44.9663       2.24583       1.00000  0.000000E+00       1.00000  0.000000E+00      0.801551       63.8888       1.00000       24.2709  0.000000E+00       1.20513  0.000000E+00       1.20513  0.000000E+00  0.000000E+00      -2.41026       45.1306       1.58302        1       Scatag_D
      22       1778.43       6600.40  0.000000E+00  0.000000E+00       2174.68  0.000000E+00       3311.25       6199.94  0.000000E+00       10175.6       53.1350       10815.4 -1.000000E+10 -1.000000E+10 -1.000000E+10 -1.000000E+10 -1.000000E+10  0.000000E+00  0.000000E+00  0.000000E+00  0.000000E+00  0.000000E+00  0.000000E+00  0.000000E+00  0.000000E+00  0.000000E+00  0.000000E+00  0.000000E+00  0.000000E+00       Funtag_D
      23  4.967215E+05  4.554908E+05       41230.7       26.7828     0.0491339      0.348786      0.215390      0.236137     0.0341337     0.0144954    0.00792812       1.10255       Opttag_D
      23       45.0023       2.24251       1.00000  0.000000E+00       1.00000  0.000000E+00      0.801292       64.0224       1.00000       24.2613  0.000000E+00       1.19623  0.000000E+00       1.19623  0.000000E+00  0.000000E+00      -2.39246       45.1306       1.58302        1       Scatag_D
      23       1772.12       6634.57  0.000000E+00  0.000000E+00       2173.86  0.000000E+00       3325.34       6187.16  0.000000E+00       10257.7       53.4840       10826.5 -1.000000E+10 -1.000000E+10 -1.000000E+10 -1.000000E+10 -1.000000E+10  0.000000E+00  0.000000E+00  0.000000E+00  0.000000E+00  0.000000E+00  0.000000E+00  0.000000E+00  0.000000E+00  0.000000E+00  0.000000E+00  0.000000E+00  0.000000E+00       Funtag_D
      24  4.965279E+05  4.549973E+05       41530.6       36.9929     0.0540473      0.403022      0.214916      0.235623     0.0353477     0.0150734    0.00791216       1.10838       Opttag_D
      24       45.0807       2.23881       1.00000  0.000000E+00       1.00000  0.000000E+00      0.800809       64.2963       1.00000       24.2441  0.000000E+00       1.17849  0.000000E+00       1.17849  0.000000E+00  0.000000E+00      -2.35698       45.1306       1.58302        1       Scatag_D
      24       1764.99       6704.93  0.000000E+00  0.000000E+00       2172.58  0.000000E+00       3360.82       6202.91  0.000000E+00       10416.9       54.5246       10852.9 -1.000000E+10 -1.000000E+10 -1.000000E+10 -1.000000E+10 -1.000000E+10  0.000000E+00  0.000000E+00  0.000000E+00  0.000000E+00  0.000000E+00  0.000000E+00  0.000000E+00  0.000000E+00  0.000000E+00  0.000000E+00  0.000000E+00  0.000000E+00       Funtag_D
      25  4.963535E+05  4.548198E+05       41533.8       23.0710     0.0557564      0.423937      0.214746      0.235422     0.0357845     0.0152905    0.00787512       1.10650       Opttag_D
      25       45.1145       2.23835       1.00000  0.000000E+00       1.00000  0.000000E+00      0.800651       64.3997       1.00000       24.2391  0.000000E+00       1.17140  0.000000E+00       1.17140  0.000000E+00  0.000000E+00      -2.34280       45.1306       1.58302        1       Scatag_D
      25       1748.51       6682.16  0.000000E+00  0.000000E+00       2172.26  0.000000E+00       3378.40       6173.08  0.000000E+00       10466.4       55.0796       10857.9 -1.000000E+10 -1.000000E+10 -1.000000E+10 -1.000000E+10 -1.000000E+10  0.000000E+00  0.000000E+00  0.000000E+00  0.000000E+00  0.000000E+00  0.000000E+00  0.000000E+00  0.000000E+00  0.000000E+00  0.000000E+00  0.000000E+00  0.000000E+00       Funtag_D
      26  4.962098E+05  4.546725E+05       41537.3       20.4647     0.0569813      0.441061      0.214606      0.235267     0.0361467     0.0154777    0.00785718       1.10638       Opttag_D
      26       45.1447       2.23828       1.00000  0.000000E+00       1.00000  0.000000E+00      0.800542       64.4785       1.00000       24.2375  0.000000E+00       1.16507  0.000000E+00       1.16507  0.000000E+00  0.000000E+00      -2.33015       45.1306       1.58302        1       Scatag_D
      26       1740.55       6680.69  0.000000E+00  0.000000E+00       2171.74  0.000000E+00       3395.11       6140.27  0.000000E+00       10494.8       55.6115       10858.5 -1.000000E+10 -1.000000E+10 -1.000000E+10 -1.000000E+10 -1.000000E+10  0.000000E+00  0.000000E+00  0.000000E+00  0.000000E+00  0.000000E+00  0.000000E+00  0.000000E+00  0.000000E+00  0.000000E+00  0.000000E+00  0.000000E+00  0.000000E+00       Funtag_D
      27  4.960584E+05  4.544306E+05       41627.8       23.8171     0.0590767      0.470084      0.214379      0.235010     0.0367416     0.0158031    0.00785763       1.10726       Opttag_D
      27       45.1956       2.23833       1.00000  0.000000E+00       1.00000  0.000000E+00      0.800369       64.6070       1.00000       24.2351  0.000000E+00       1.15374  0.000000E+00       1.15374  0.000000E+00  0.000000E+00      -2.30748       45.1306       1.58302        1       Scatag_D
      27       1740.75       6691.37  0.000000E+00  0.000000E+00       2170.98  0.000000E+00       3424.49       6135.17  0.000000E+00       10544.4       56.2569       10864.4 -1.000000E+10 -1.000000E+10 -1.000000E+10 -1.000000E+10 -1.000000E+10  0.000000E+00  0.000000E+00  0.000000E+00  0.000000E+00  0.000000E+00  0.000000E+00  0.000000E+00  0.000000E+00  0.000000E+00  0.000000E+00  0.000000E+00  0.000000E+00       Funtag_D
      28  4.959625E+05  4.539897E+05       41972.8       43.7734     0.0634600      0.529081      0.213952      0.234526     0.0378263     0.0163812    0.00792100       1.11425       Opttag_D
      28       45.2979       2.23976       1.00000  0.000000E+00       1.00000  0.000000E+00      0.800050       64.8654       1.00000       24.2305  0.000000E+00       1.13069  0.000000E+00       1.13069  0.000000E+00  0.000000E+00      -2.26138       45.1306       1.58302        1       Scatag_D
      28       1768.94       6776.12  0.000000E+00  0.000000E+00       2169.58  0.000000E+00       3485.22       6175.37  0.000000E+00       10652.7       57.1464       10887.8 -1.000000E+10 -1.000000E+10 -1.000000E+10 -1.000000E+10 -1.000000E+10  0.000000E+00  0.000000E+00  0.000000E+00  0.000000E+00  0.000000E+00  0.000000E+00  0.000000E+00  0.000000E+00  0.000000E+00  0.000000E+00  0.000000E+00  0.000000E+00       Funtag_D
      29  4.957996E+05  4.539270E+05       41872.6       18.9898     0.0639648      0.537286      0.213892      0.234463     0.0379804     0.0165047    0.00789564       1.10844       Opttag_D
      29       45.3111       2.23974       1.00000  0.000000E+00       1.00000  0.000000E+00      0.800017       64.8936       1.00000       24.2302  0.000000E+00       1.12707  0.000000E+00       1.12707  0.000000E+00  0.000000E+00      -2.25415       45.1306       1.58302        1       Scatag_D
      29       1757.63       6705.68  0.000000E+00  0.000000E+00       2169.45  0.000000E+00       3492.35       6143.04  0.000000E+00       10660.5       56.9445       10887.1 -1.000000E+10 -1.000000E+10 -1.000000E+10 -1.000000E+10 -1.000000E+10  0.000000E+00  0.000000E+00  0.000000E+00  0.000000E+00  0.000000E+00  0.000000E+00  0.000000E+00  0.000000E+00  0.000000E+00  0.000000E+00  0.000000E+00  0.000000E+00       Funtag_D
      30  4.957075E+05  4.538278E+05       41879.7       16.0486     0.0648502      0.550696      0.213799      0.234367     0.0382246     0.0166744    0.00790317       1.10862       Opttag_D
      30       45.3315       2.23960       1.00000  0.000000E+00       1.00000  0.000000E+00      0.799959       64.9406       1.00000       24.2298  0.000000E+00       1.12156  0.000000E+00       1.12156  0.000000E+00  0.000000E+00      -2.24312       45.1306       1.58302        1       Scatag_D
      30       1760.98       6707.83  0.000000E+00  0.000000E+00       2169.21  0.000000E+00       3502.51       6115.87  0.000000E+00       10678.0       56.7520       10888.6 -1.000000E+10 -1.000000E+10 -1.000000E+10 -1.000000E+10 -1.000000E+10  0.000000E+00  0.000000E+00  0.000000E+00  0.000000E+00  0.000000E+00  0.000000E+00  0.000000E+00  0.000000E+00  0.000000E+00  0.000000E+00  0.000000E+00  0.000000E+00       Funtag_D
      31  4.956018E+05  4.536092E+05       41992.7       18.5116     0.0670204      0.582662      0.213587      0.234136     0.0387622     0.0170149    0.00792725       1.10983       Opttag_D
      31       45.3819       2.24016       1.00000  0.000000E+00       1.00000  0.000000E+00      0.799823       65.0611       1.00000       24.2284  0.000000E+00       1.10904  0.000000E+00       1.10904  0.000000E+00  0.000000E+00      -2.21807       45.1306       1.58302        1       Scatag_D
      31       1771.73       6722.43  0.000000E+00  0.000000E+00       2168.92  0.000000E+00       3528.56       6123.47  0.000000E+00       10723.9       56.8849       10896.7 -1.000000E+10 -1.000000E+10 -1.000000E+10 -1.000000E+10 -1.000000E+10  0.000000E+00  0.000000E+00  0.000000E+00  0.000000E+00  0.000000E+00  0.000000E+00  0.000000E+00  0.000000E+00  0.000000E+00  0.000000E+00  0.000000E+00  0.000000E+00       Funtag_D
      32  4.955205E+05  4.532437E+05       42276.9       40.4141     0.0708281      0.641123      0.213232      0.233759     0.0396614     0.0175642    0.00799130       1.11506       Opttag_D
      32       45.4703       2.24163       1.00000  0.000000E+00       1.00000  0.000000E+00      0.799603       65.2683       1.00000       24.2271  0.000000E+00       1.08740  0.000000E+00       1.08740  0.000000E+00  0.000000E+00      -2.17480       45.1306       1.58302        1       Scatag_D
      32       1800.48       6785.95  0.000000E+00  0.000000E+00       2169.09  0.000000E+00       3570.94       6181.48  0.000000E+00       10799.5       57.5176       10911.9 -1.000000E+10 -1.000000E+10 -1.000000E+10 -1.000000E+10 -1.000000E+10  0.000000E+00  0.000000E+00  0.000000E+00  0.000000E+00  0.000000E+00  0.000000E+00  0.000000E+00  0.000000E+00  0.000000E+00  0.000000E+00  0.000000E+00  0.000000E+00       Funtag_D
      33  4.953837E+05  4.531177E+05       42266.0       17.7384     0.0723151      0.664128      0.213104      0.233613     0.0399712     0.0177700    0.00797711       1.11168       Opttag_D
      33       45.5048       2.24281       1.00000  0.000000E+00       1.00000  0.000000E+00      0.799526       65.3508       1.00000       24.2250  0.000000E+00       1.07951  0.000000E+00       1.07951  0.000000E+00  0.000000E+00      -2.15901       45.1306       1.58302        1       Scatag_D
      33       1794.09       6744.89  0.000000E+00  0.000000E+00       2169.41  0.000000E+00       3586.85       6168.43  0.000000E+00       10828.8       57.8300       10915.7 -1.000000E+10 -1.000000E+10 -1.000000E+10 -1.000000E+10 -1.000000E+10  0.000000E+00  0.000000E+00  0.000000E+00  0.000000E+00  0.000000E+00  0.000000E+00  0.000000E+00  0.000000E+00  0.000000E+00  0.000000E+00  0.000000E+00  0.000000E+00       Funtag_D
      34  4.952994E+05  4.530255E+05       42273.9       13.8687     0.0734296      0.683508      0.213011      0.233500     0.0401980     0.0179429    0.00797127       1.11165       Opttag_D
      34       45.5308       2.24380       1.00000  0.000000E+00       1.00000  0.000000E+00      0.799471       65.4120       1.00000       24.2214  0.000000E+00       1.07389  0.000000E+00       1.07389  0.000000E+00  0.000000E+00      -2.14777       45.1306       1.58302        1       Scatag_D
      34       1791.46       6744.51  0.000000E+00  0.000000E+00       2169.82  0.000000E+00       3595.56       6152.64  0.000000E+00       10846.0       58.0887       10915.8 -1.000000E+10 -1.000000E+10 -1.000000E+10 -1.000000E+10 -1.000000E+10  0.000000E+00  0.000000E+00  0.000000E+00  0.000000E+00  0.000000E+00  0.000000E+00  0.000000E+00  0.000000E+00  0.000000E+00  0.000000E+00  0.000000E+00  0.000000E+00       Funtag_D
      35  4.952100E+05  4.528933E+05       42316.7       16.6587     0.0752751      0.715936      0.212864      0.233312     0.0405231     0.0182175    0.00796548       1.11217       Opttag_D
      35       45.5729       2.24593       1.00000  0.000000E+00       1.00000  0.000000E+00      0.799385       65.5109       1.00000       24.2140  0.000000E+00       1.06510  0.000000E+00       1.06510  0.000000E+00  0.000000E+00      -2.13020       45.1306       1.58302        1       Scatag_D
      35       1788.86       6750.83  0.000000E+00  0.000000E+00       2170.73  0.000000E+00       3608.98       6148.06  0.000000E+00       10874.4       58.2926       10916.5 -1.000000E+10 -1.000000E+10 -1.000000E+10 -1.000000E+10 -1.000000E+10  0.000000E+00  0.000000E+00  0.000000E+00  0.000000E+00  0.000000E+00  0.000000E+00  0.000000E+00  0.000000E+00  0.000000E+00  0.000000E+00  0.000000E+00  0.000000E+00       Funtag_D
      36  4.951155E+05  4.527080E+05       42407.5       32.7945     0.0786392      0.776268      0.212643      0.232980     0.0409790     0.0186549    0.00796156       1.11430       Opttag_D
      36       45.6461       2.25094       1.00000  0.000000E+00       1.00000  0.000000E+00      0.799235       65.6799       1.00000       24.1978  0.000000E+00       1.05119  0.000000E+00       1.05119  0.000000E+00  0.000000E+00      -2.10237       45.1306       1.58302        1       Scatag_D
      36       1787.10       6776.75  0.000000E+00  0.000000E+00       2173.38  0.000000E+00       3628.68       6141.77  0.000000E+00       10924.8       58.0733       10917.0 -1.000000E+10 -1.000000E+10 -1.000000E+10 -1.000000E+10 -1.000000E+10  0.000000E+00  0.000000E+00  0.000000E+00  0.000000E+00  0.000000E+00  0.000000E+00  0.000000E+00  0.000000E+00  0.000000E+00  0.000000E+00  0.000000E+00  0.000000E+00       Funtag_D
      37  4.950030E+05  4.526040E+05       42399.0       16.2637     0.0808834      0.816463      0.212512      0.232770     0.0412349     0.0189200    0.00793796       1.11249       Opttag_D
      37       45.6932       2.25457       1.00000  0.000000E+00       1.00000  0.000000E+00      0.799135       65.7878       1.00000       24.1882  0.000000E+00       1.04260  0.000000E+00       1.04260  0.000000E+00  0.000000E+00      -2.08520       45.1306       1.58302        1       Scatag_D
      37       1776.52       6754.70  0.000000E+00  0.000000E+00       2175.22  0.000000E+00       3640.97       6115.30  0.000000E+00       10959.6       58.0244       10918.7 -1.000000E+10 -1.000000E+10 -1.000000E+10 -1.000000E+10 -1.000000E+10  0.000000E+00  0.000000E+00  0.000000E+00  0.000000E+00  0.000000E+00  0.000000E+00  0.000000E+00  0.000000E+00  0.000000E+00  0.000000E+00  0.000000E+00  0.000000E+00       Funtag_D
      38  4.949342E+05  4.525720E+05       42362.2       12.1979     0.0816753      0.833144      0.212471      0.232689     0.0413135     0.0190136    0.00792307       1.11196       Opttag_D
      38       45.7071       2.25547       1.00000  0.000000E+00       1.00000  0.000000E+00      0.799090       65.8143       1.00000       24.1837  0.000000E+00       1.04079  0.000000E+00       1.04079  0.000000E+00  0.000000E+00      -2.08158       45.1306       1.58302        1       Scatag_D
      38       1769.86       6748.28  0.000000E+00  0.000000E+00       2176.01  0.000000E+00       3639.56       6087.89  0.000000E+00       10967.8       57.8724       10915.0 -1.000000E+10 -1.000000E+10 -1.000000E+10 -1.000000E+10 -1.000000E+10  0.000000E+00  0.000000E+00  0.000000E+00  0.000000E+00  0.000000E+00  0.000000E+00  0.000000E+00  0.000000E+00  0.000000E+00  0.000000E+00  0.000000E+00  0.000000E+00       Funtag_D
      39  4.948510E+05  4.525019E+05       42349.1       16.2146     0.0832101      0.865766      0.212387      0.232529     0.0414860     0.0191745    0.00791301       1.11171       Opttag_D
      39       45.7331       2.25673       1.00000  0.000000E+00       1.00000  0.000000E+00      0.799003       65.8609       1.00000       24.1772  0.000000E+00       1.03749  0.000000E+00       1.03749  0.000000E+00  0.000000E+00      -2.07498       45.1306       1.58302        1       Scatag_D
      39       1765.37       6745.27  0.000000E+00  0.000000E+00       2177.62  0.000000E+00       3636.89       6072.32  0.000000E+00       10983.8       57.8115       10910.0 -1.000000E+10 -1.000000E+10 -1.000000E+10 -1.000000E+10 -1.000000E+10  0.000000E+00  0.000000E+00  0.000000E+00  0.000000E+00  0.000000E+00  0.000000E+00  0.000000E+00  0.000000E+00  0.000000E+00  0.000000E+00  0.000000E+00  0.000000E+00       Funtag_D
      40  4.947928E+05  4.523832E+05       42409.6       25.8439     0.0856057      0.915573      0.212261      0.232300     0.0417779     0.0193655    0.00792015       1.11213       Opttag_D
      40       45.7731       2.25822       1.00000  0.000000E+00       1.00000  0.000000E+00      0.798880       65.9268       1.00000       24.1717  0.000000E+00       1.03249  0.000000E+00       1.03249  0.000000E+00  0.000000E+00      -2.06498       45.1306       1.58302        1       Scatag_D
      40       1768.56       6750.36  0.000000E+00  0.000000E+00       2179.95  0.000000E+00       3636.90       6096.86  0.000000E+00       11010.2       57.9861       10908.8 -1.000000E+10 -1.000000E+10 -1.000000E+10 -1.000000E+10 -1.000000E+10  0.000000E+00  0.000000E+00  0.000000E+00  0.000000E+00  0.000000E+00  0.000000E+00  0.000000E+00  0.000000E+00  0.000000E+00  0.000000E+00  0.000000E+00  0.000000E+00       Funtag_D
      41  4.947171E+05  4.523405E+05       42376.7       14.0057     0.0861640      0.928563      0.212223      0.232248     0.0418831     0.0194024    0.00792224       1.11077       Opttag_D
      41       45.7809       2.25772       1.00000  0.000000E+00       1.00000  0.000000E+00      0.798842       65.9349       1.00000       24.1722  0.000000E+00       1.03176  0.000000E+00       1.03176  0.000000E+00  0.000000E+00      -2.06351       45.1306       1.58302        1       Scatag_D
      41       1769.49       6733.87  0.000000E+00  0.000000E+00       2180.50  0.000000E+00       3635.87       6076.50  0.000000E+00       11014.6       58.0617       10907.8 -1.000000E+10 -1.000000E+10 -1.000000E+10 -1.000000E+10 -1.000000E+10  0.000000E+00  0.000000E+00  0.000000E+00  0.000000E+00  0.000000E+00  0.000000E+00  0.000000E+00  0.000000E+00  0.000000E+00  0.000000E+00  0.000000E+00  0.000000E+00       Funtag_D
      42  4.946471E+05  4.522602E+05       42386.9       12.0584     0.0872036      0.951943      0.212153      0.232160     0.0420804     0.0194618    0.00793750       1.11025       Opttag_D
      42       45.7959       2.25687       1.00000  0.000000E+00       1.00000  0.000000E+00      0.798778       65.9509       1.00000       24.1742  0.000000E+00       1.03028  0.000000E+00       1.03028  0.000000E+00  0.000000E+00      -2.06055       45.1306       1.58302        1       Scatag_D
      42       1776.32       6727.51  0.000000E+00  0.000000E+00       2181.34  0.000000E+00       3636.58       6075.73  0.000000E+00       11023.7       58.1282       10907.6 -1.000000E+10 -1.000000E+10 -1.000000E+10 -1.000000E+10 -1.000000E+10  0.000000E+00  0.000000E+00  0.000000E+00  0.000000E+00  0.000000E+00  0.000000E+00  0.000000E+00  0.000000E+00  0.000000E+00  0.000000E+00  0.000000E+00  0.000000E+00       Funtag_D
      43  4.945884E+05  4.521795E+05       42408.9       13.7058     0.0885553      0.981941      0.212081      0.232064     0.0422791     0.0195193    0.00795195       1.10995       Opttag_D
      43       45.8153       2.25656       1.00000  0.000000E+00       1.00000  0.000000E+00      0.798701       65.9736       1.00000       24.1757  0.000000E+00       1.02852  0.000000E+00       1.02852  0.000000E+00  0.000000E+00      -2.05703       45.1306       1.58302        1       Scatag_D
      43       1782.79       6723.88  0.000000E+00  0.000000E+00       2182.40  0.000000E+00       3639.10       6078.00  0.000000E+00       11036.2       57.9229       10908.6 -1.000000E+10 -1.000000E+10 -1.000000E+10 -1.000000E+10 -1.000000E+10  0.000000E+00  0.000000E+00  0.000000E+00  0.000000E+00  0.000000E+00  0.000000E+00  0.000000E+00  0.000000E+00  0.000000E+00  0.000000E+00  0.000000E+00  0.000000E+00       Funtag_D
      44  4.945521E+05  4.520205E+05       42531.6       33.4690     0.0918044       1.05434      0.211934      0.231898     0.0426700     0.0195766    0.00800031       1.11127       Opttag_D
      44       45.8588       2.25629       1.00000  0.000000E+00       1.00000  0.000000E+00      0.798517       66.0129       1.00000       24.1857  0.000000E+00       1.02540  0.000000E+00       1.02540  0.000000E+00  0.000000E+00      -2.05079       45.1306       1.58302        1       Scatag_D
      44       1804.54       6739.87  0.000000E+00  0.000000E+00       2185.01  0.000000E+00       3648.43       6116.55  0.000000E+00       11066.6       57.1131       10913.5 -1.000000E+10 -1.000000E+10 -1.000000E+10 -1.000000E+10 -1.000000E+10  0.000000E+00  0.000000E+00  0.000000E+00  0.000000E+00  0.000000E+00  0.000000E+00  0.000000E+00  0.000000E+00  0.000000E+00  0.000000E+00  0.000000E+00  0.000000E+00       Funtag_D
      45  4.944556E+05  4.520045E+05       42451.1       12.0681     0.0923184       1.06606      0.211919      0.231877     0.0427095     0.0195878    0.00797692       1.10927       Opttag_D
      45       45.8654       2.25649       1.00000  0.000000E+00       1.00000  0.000000E+00      0.798484       66.0189       1.00000       24.1877  0.000000E+00       1.02484  0.000000E+00       1.02484  0.000000E+00  0.000000E+00      -2.04968       45.1306       1.58302        1       Scatag_D
      45       1794.00       6715.66  0.000000E+00  0.000000E+00       2185.23  0.000000E+00       3650.39       6065.35  0.000000E+00       11070.6       56.9502       10913.0 -1.000000E+10 -1.000000E+10 -1.000000E+10 -1.000000E+10 -1.000000E+10  0.000000E+00  0.000000E+00  0.000000E+00  0.000000E+00  0.000000E+00  0.000000E+00  0.000000E+00  0.000000E+00  0.000000E+00  0.000000E+00  0.000000E+00  0.000000E+00       Funtag_D
      46  4.944176E+05  4.519967E+05       42420.8       9.43485     0.0925666       1.07295      0.211917      0.231874     0.0427286     0.0195910    0.00796326       1.10903       Opttag_D
      46       45.8674       2.25630       1.00000  0.000000E+00       1.00000  0.000000E+00      0.798457       66.0150       1.00000       24.1914  0.000000E+00       1.02475  0.000000E+00       1.02475  0.000000E+00  0.000000E+00      -2.04949       45.1306       1.58302        1       Scatag_D
      46       1787.86       6712.78  0.000000E+00  0.000000E+00       2185.21  0.000000E+00       3651.54       6045.15  0.000000E+00       11070.8       56.8140       10910.7 -1.000000E+10 -1.000000E+10 -1.000000E+10 -1.000000E+10 -1.000000E+10  0.000000E+00  0.000000E+00  0.000000E+00  0.000000E+00  0.000000E+00  0.000000E+00  0.000000E+00  0.000000E+00  0.000000E+00  0.000000E+00  0.000000E+00  0.000000E+00       Funtag_D
      47  4.943610E+05  4.519550E+05       42406.0       12.4422     0.0936785       1.10036      0.211888      0.231837     0.0428312     0.0196032    0.00794839       1.10882       Opttag_D
      47       45.8793       2.25591       1.00000  0.000000E+00       1.00000  0.000000E+00      0.798369       66.0119       1.00000       24.2036  0.000000E+00       1.02391  0.000000E+00       1.02391  0.000000E+00  0.000000E+00      -2.04781       45.1306       1.58302        1       Scatag_D
      47       1781.19       6710.27  0.000000E+00  0.000000E+00       2185.72  0.000000E+00       3655.87       6031.29  0.000000E+00       11077.2       56.6285       10907.9 -1.000000E+10 -1.000000E+10 -1.000000E+10 -1.000000E+10 -1.000000E+10  0.000000E+00  0.000000E+00  0.000000E+00  0.000000E+00  0.000000E+00  0.000000E+00  0.000000E+00  0.000000E+00  0.000000E+00  0.000000E+00  0.000000E+00  0.000000E+00       Funtag_D
      48  4.942950E+05  4.518835E+05       42411.5       20.8144     0.0956138       1.14780      0.211845      0.231768     0.0430071     0.0196372    0.00792949       1.10885       Opttag_D
      48       45.9005       2.25531       1.00000  0.000000E+00       1.00000  0.000000E+00      0.798217       66.0069       1.00000       24.2285  0.000000E+00       1.02213  0.000000E+00       1.02213  0.000000E+00  0.000000E+00      -2.04425       45.1306       1.58302        1       Scatag_D
      48       1772.73       6710.57  0.000000E+00  0.000000E+00       2186.78  0.000000E+00       3662.62       6030.55  0.000000E+00       11088.1       56.6784       10903.5 -1.000000E+10 -1.000000E+10 -1.000000E+10 -1.000000E+10 -1.000000E+10  0.000000E+00  0.000000E+00  0.000000E+00  0.000000E+00  0.000000E+00  0.000000E+00  0.000000E+00  0.000000E+00  0.000000E+00  0.000000E+00  0.000000E+00  0.000000E+00       Funtag_D
      49  4.942242E+05  4.518181E+05       42406.1       13.4007     0.0979126       1.20171      0.211808      0.231687     0.0431680     0.0196510    0.00791231       1.10818       Opttag_D
      49       45.9260       2.25537       1.00000  0.000000E+00       1.00000  0.000000E+00      0.798059       66.0059       1.00000       24.2567  0.000000E+00       1.02035  0.000000E+00       1.02035  0.000000E+00  0.000000E+00      -2.04070       45.1306       1.58302        1       Scatag_D
      49       1765.06       6702.47  0.000000E+00  0.000000E+00       2188.38  0.000000E+00       3670.65       6016.81  0.000000E+00       11103.9       56.6981       10902.1 -1.000000E+10 -1.000000E+10 -1.000000E+10 -1.000000E+10 -1.000000E+10  0.000000E+00  0.000000E+00  0.000000E+00  0.000000E+00  0.000000E+00  0.000000E+00  0.000000E+00  0.000000E+00  0.000000E+00  0.000000E+00  0.000000E+00  0.000000E+00       Funtag_D
      50  4.941745E+05  4.517933E+05       42381.2       9.58527     0.0989027       1.22525      0.211797      0.231651     0.0432290     0.0196465    0.00790236       1.10778       Opttag_D
      50       45.9371       2.25554       1.00000  0.000000E+00       1.00000  0.000000E+00      0.797993       66.0074       1.00000       24.2690  0.000000E+00       1.01966  0.000000E+00       1.01966  0.000000E+00  0.000000E+00      -2.03932       45.1306       1.58302        1       Scatag_D
      50       1760.62       6697.60  0.000000E+00  0.000000E+00       2189.14  0.000000E+00       3672.09       5996.88  0.000000E+00       11108.9       56.6685       10899.2 -1.000000E+10 -1.000000E+10 -1.000000E+10 -1.000000E+10 -1.000000E+10  0.000000E+00  0.000000E+00  0.000000E+00  0.000000E+00  0.000000E+00  0.000000E+00  0.000000E+00  0.000000E+00  0.000000E+00  0.000000E+00  0.000000E+00  0.000000E+00       Funtag_D
      51  4.941150E+05  4.517560E+05       42359.0       13.2412      0.100618       1.26598      0.211782      0.231575     0.0433207     0.0196760    0.00789196       1.10746       Opttag_D
      51       45.9563       2.25612       1.00000  0.000000E+00       1.00000  0.000000E+00      0.797882       66.0131       1.00000       24.2895  0.000000E+00       1.01847  0.000000E+00       1.01847  0.000000E+00  0.000000E+00      -2.03694       45.1306       1.58302        1       Scatag_D
      51       1755.99       6693.73  0.000000E+00  0.000000E+00       2190.57  0.000000E+00       3672.83       5978.71  0.000000E+00       11116.2       56.5722       10894.4 -1.000000E+10 -1.000000E+10 -1.000000E+10 -1.000000E+10 -1.000000E+10  0.000000E+00  0.000000E+00  0.000000E+00  0.000000E+00  0.000000E+00  0.000000E+00  0.000000E+00  0.000000E+00  0.000000E+00  0.000000E+00  0.000000E+00  0.000000E+00       Funtag_D
      52  4.940770E+05  4.517338E+05       42343.2       15.4302      0.102130       1.30104      0.211774      0.231548     0.0433754     0.0196249    0.00788487       1.10723       Opttag_D
      52       45.9724       2.25689       1.00000  0.000000E+00       1.00000  0.000000E+00      0.797788       66.0180       1.00000       24.3061  0.000000E+00       1.01758  0.000000E+00       1.01758  0.000000E+00  0.000000E+00      -2.03516       45.1306       1.58302        1       Scatag_D
      52       1752.84       6690.98  0.000000E+00  0.000000E+00       2191.81  0.000000E+00       3673.48       5962.86  0.000000E+00       11122.7       56.5330       10892.0 -1.000000E+10 -1.000000E+10 -1.000000E+10 -1.000000E+10 -1.000000E+10  0.000000E+00  0.000000E+00  0.000000E+00  0.000000E+00  0.000000E+00  0.000000E+00  0.000000E+00  0.000000E+00  0.000000E+00  0.000000E+00  0.000000E+00  0.000000E+00       Funtag_D
      53  4.940374E+05  4.517258E+05       42311.6       10.9378      0.102449       1.30877      0.211772      0.231540     0.0433951     0.0196354    0.00788277       1.10697       Opttag_D
      53       45.9752       2.25690       1.00000  0.000000E+00       1.00000  0.000000E+00      0.797765       66.0179       1.00000       24.3089  0.000000E+00       1.01740  0.000000E+00       1.01740  0.000000E+00  0.000000E+00      -2.03480       45.1306       1.58302        1       Scatag_D
      53       1751.91       6687.86  0.000000E+00  0.000000E+00       2192.06  0.000000E+00       3672.23       5937.92  0.000000E+00       11122.2       56.5411       10890.8 -1.000000E+10 -1.000000E+10 -1.000000E+10 -1.000000E+10 -1.000000E+10  0.000000E+00  0.000000E+00  0.000000E+00  0.000000E+00  0.000000E+00  0.000000E+00  0.000000E+00  0.000000E+00  0.000000E+00  0.000000E+00  0.000000E+00  0.000000E+00       Funtag_D
      54  4.939641E+05  4.516909E+05       42273.2       10.6022      0.103981       1.34372      0.211760      0.231520     0.0434808     0.0196315    0.00788638       1.10660       Opttag_D
      54       45.9889       2.25690       1.00000  0.000000E+00       1.00000  0.000000E+00      0.797660       66.0134       1.00000       24.3261  0.000000E+00       1.01640  0.000000E+00       1.01640  0.000000E+00  0.000000E+00      -2.03280       45.1306       1.58302        1       Scatag_D
      54       1753.51       6683.42  0.000000E+00  0.000000E+00       2193.41  0.000000E+00       3670.66       5900.32  0.000000E+00       11125.3       56.7715       10889.8 -1.000000E+10 -1.000000E+10 -1.000000E+10 -1.000000E+10 -1.000000E+10  0.000000E+00  0.000000E+00  0.000000E+00  0.000000E+00  0.000000E+00  0.000000E+00  0.000000E+00  0.000000E+00  0.000000E+00  0.000000E+00  0.000000E+00  0.000000E+00       Funtag_D
      55  4.939267E+05  4.516534E+05       42273.3       15.9693      0.105516       1.37711      0.211748      0.231500     0.0435731     0.0196328    0.00789090       1.10642       Opttag_D
      55       46.0018       2.25657       1.00000  0.000000E+00       1.00000  0.000000E+00      0.797554       66.0040       1.00000       24.3476  0.000000E+00       1.01560  0.000000E+00       1.01560  0.000000E+00  0.000000E+00      -2.03120       45.1306       1.58302        1       Scatag_D
      55       1755.52       6681.17  0.000000E+00  0.000000E+00       2194.70  0.000000E+00       3670.84       5894.41  0.000000E+00       11130.0       57.1627       10889.5 -1.000000E+10 -1.000000E+10 -1.000000E+10 -1.000000E+10 -1.000000E+10  0.000000E+00  0.000000E+00  0.000000E+00  0.000000E+00  0.000000E+00  0.000000E+00  0.000000E+00  0.000000E+00  0.000000E+00  0.000000E+00  0.000000E+00  0.000000E+00       Funtag_D
      56  4.938805E+05  4.516213E+05       42259.3       11.4775      0.106724       1.40240      0.211741      0.231496     0.0436521     0.0196057    0.00789515       1.10612       Opttag_D
      56       46.0113       2.25608       1.00000  0.000000E+00       1.00000  0.000000E+00      0.797471       65.9927       1.00000       24.3664  0.000000E+00       1.01485  0.000000E+00       1.01485  0.000000E+00  0.000000E+00      -2.02970       45.1306       1.58302        1       Scatag_D
      56       1757.41       6677.59  0.000000E+00  0.000000E+00       2195.76  0.000000E+00       3671.12       5876.69  0.000000E+00       11133.8       57.4941       10889.4 -1.000000E+10 -1.000000E+10 -1.000000E+10 -1.000000E+10 -1.000000E+10  0.000000E+00  0.000000E+00  0.000000E+00  0.000000E+00  0.000000E+00  0.000000E+00  0.000000E+00  0.000000E+00  0.000000E+00  0.000000E+00  0.000000E+00  0.000000E+00       Funtag_D
      57  4.938181E+05  4.515598E+05       42258.4       11.4919      0.109184       1.45116      0.211733      0.231495     0.0438035     0.0195637    0.00790069       1.10572       Opttag_D
      57       46.0293       2.25491       1.00000  0.000000E+00       1.00000  0.000000E+00      0.797303       65.9667       1.00000       24.4080  0.000000E+00       1.01372  0.000000E+00       1.01372  0.000000E+00  0.000000E+00      -2.02743       45.1306       1.58302        1       Scatag_D
      57       1759.88       6672.83  0.000000E+00  0.000000E+00       2197.82  0.000000E+00       3671.27       5869.41  0.000000E+00       11140.6       58.0459       10888.5 -1.000000E+10 -1.000000E+10 -1.000000E+10 -1.000000E+10 -1.000000E+10  0.000000E+00  0.000000E+00  0.000000E+00  0.000000E+00  0.000000E+00  0.000000E+00  0.000000E+00  0.000000E+00  0.000000E+00  0.000000E+00  0.000000E+00  0.000000E+00       Funtag_D
      58  4.937856E+05  4.515307E+05       42254.9       13.4242      0.110625       1.47765      0.211736      0.231518     0.0438749     0.0195067    0.00789965       1.10545       Opttag_D
      58       46.0391       2.25432       1.00000  0.000000E+00       1.00000  0.000000E+00      0.797205       65.9511       1.00000       24.4336  0.000000E+00       1.01341  0.000000E+00       1.01341  0.000000E+00  0.000000E+00      -2.02682       45.1306       1.58302        1       Scatag_D
      58       1759.42       6669.52  0.000000E+00  0.000000E+00       2198.91  0.000000E+00       3670.26       5868.84  0.000000E+00       11143.3       58.1651       10886.5 -1.000000E+10 -1.000000E+10 -1.000000E+10 -1.000000E+10 -1.000000E+10  0.000000E+00  0.000000E+00  0.000000E+00  0.000000E+00  0.000000E+00  0.000000E+00  0.000000E+00  0.000000E+00  0.000000E+00  0.000000E+00  0.000000E+00  0.000000E+00       Funtag_D
      59  4.937556E+05  4.515214E+05       42234.2       9.65472      0.111010       1.48356      0.211733      0.231524     0.0438977     0.0194952    0.00789345       1.10522       Opttag_D
      59       46.0412       2.25399       1.00000  0.000000E+00       1.00000  0.000000E+00      0.797173       65.9466       1.00000       24.4416  0.000000E+00       1.01350  0.000000E+00       1.01350  0.000000E+00  0.000000E+00      -2.02699       45.1306       1.58302        1       Scatag_D
      59       1756.65       6666.76  0.000000E+00  0.000000E+00       2199.01  0.000000E+00       3669.10       5858.16  0.000000E+00       11142.1       58.0915       10884.3 -1.000000E+10 -1.000000E+10 -1.000000E+10 -1.000000E+10 -1.000000E+10  0.000000E+00  0.000000E+00  0.000000E+00  0.000000E+00  0.000000E+00  0.000000E+00  0.000000E+00  0.000000E+00  0.000000E+00  0.000000E+00  0.000000E+00  0.000000E+00       Funtag_D

 An atom has moved more than     1.50 A from the initial/last
 position - if this was an accepted trial point, probably need to update the
 non-bonded contact list.

      60  4.937001E+05  4.514956E+05       42204.5       9.80167      0.112266       1.50144      0.211733      0.231546     0.0439612     0.0194782    0.00787648       1.10477       Opttag_D
      60       46.0479       2.25306       1.00000  0.000000E+00       1.00000  0.000000E+00      0.797068       65.9328       1.00000       24.4678  0.000000E+00       1.01388  0.000000E+00       1.01388  0.000000E+00  0.000000E+00      -2.02776       45.1306       1.58302        1       Scatag_D
      60       1749.11       6661.36  0.000000E+00  0.000000E+00       2199.23  0.000000E+00       3666.08       5855.32  0.000000E+00       11137.7       57.9804       10877.7 -1.000000E+10 -1.000000E+10 -1.000000E+10 -1.000000E+10 -1.000000E+10  0.000000E+00  0.000000E+00  0.000000E+00  0.000000E+00  0.000000E+00  0.000000E+00  0.000000E+00  0.000000E+00  0.000000E+00  0.000000E+00  0.000000E+00  0.000000E+00       Funtag_D

 An atom has moved more than     1.50 A from the initial/last
 position - need to update the non-bonded contact list.


 ############################################################################################################
 ###################################################### setting up contact terms
 ############################################################################################################

      61  4.936692E+05  4.514760E+05       42193.2       12.8738      0.113443       1.51841      0.211737      0.231579     0.0440095     0.0194099    0.00787063       1.10444       Opttag_D
      61       46.0549       2.25241       1.00000  0.000000E+00       1.00000  0.000000E+00      0.796978       65.9188       1.00000       24.4925  0.000000E+00       1.01397  0.000000E+00       1.01397  0.000000E+00  0.000000E+00      -2.02795       45.1306       1.58302        1       Scatag_D
      61       1746.51       6657.33  0.000000E+00  0.000000E+00       2199.77  0.000000E+00       3665.93       5855.84  0.000000E+00       11136.1       58.1632       10873.6 -1.000000E+10 -1.000000E+10 -1.000000E+10 -1.000000E+10 -1.000000E+10  0.000000E+00  0.000000E+00  0.000000E+00  0.000000E+00  0.000000E+00  0.000000E+00  0.000000E+00  0.000000E+00  0.000000E+00  0.000000E+00  0.000000E+00  0.000000E+00       Funtag_D
      62  4.936300E+05  4.514584E+05       42171.6       11.0051      0.114656       1.53485      0.211743      0.231613     0.0440526     0.0193679    0.00786613       1.10409       Opttag_D
      62       46.0620       2.25185       1.00000  0.000000E+00       1.00000  0.000000E+00      0.796885       65.9047       1.00000       24.5179  0.000000E+00       1.01407  0.000000E+00       1.01407  0.000000E+00  0.000000E+00      -2.02814       45.1306       1.58302        1       Scatag_D
      62       1744.52       6653.13  0.000000E+00  0.000000E+00       2200.35  0.000000E+00       3666.97       5844.75  0.000000E+00       11134.1       58.3985       10869.4 -1.000000E+10 -1.000000E+10 -1.000000E+10 -1.000000E+10 -1.000000E+10  0.000000E+00  0.000000E+00  0.000000E+00  0.000000E+00  0.000000E+00  0.000000E+00  0.000000E+00  0.000000E+00  0.000000E+00  0.000000E+00  0.000000E+00  0.000000E+00       Funtag_D
      63  4.935904E+05  4.514221E+05       42168.4       21.6236      0.118198       1.57862      0.211769      0.231697     0.0441421     0.0193447    0.00786420       1.10371       Opttag_D
      63       46.0820       2.25042       1.00000  0.000000E+00       1.00000  0.000000E+00      0.796627       65.8593       1.00000       24.5929  0.000000E+00       1.01545  0.000000E+00       1.01545  0.000000E+00  0.000000E+00      -2.03089       45.1306       1.58302        1       Scatag_D
      63       1743.66       6648.57  0.000000E+00  0.000000E+00       2202.35  0.000000E+00       3674.99       5853.27  0.000000E+00       11128.4       58.9970       10858.1 -1.000000E+10 -1.000000E+10 -1.000000E+10 -1.000000E+10 -1.000000E+10  0.000000E+00  0.000000E+00  0.000000E+00  0.000000E+00  0.000000E+00  0.000000E+00  0.000000E+00  0.000000E+00  0.000000E+00  0.000000E+00  0.000000E+00  0.000000E+00       Funtag_D
      64  4.935390E+05  4.514152E+05       42123.8       9.04463      0.118798       1.58452      0.211775      0.231714     0.0441590     0.0193285    0.00786553       1.10307       Opttag_D
      64       46.0850       2.25020       1.00000  0.000000E+00       1.00000  0.000000E+00      0.796577       65.8530       1.00000       24.6045  0.000000E+00       1.01546  0.000000E+00       1.01546  0.000000E+00  0.000000E+00      -2.03093       45.1306       1.58302        1       Scatag_D
      64       1744.25       6640.84  0.000000E+00  0.000000E+00       2202.81  0.000000E+00       3677.64       5817.57  0.000000E+00       11126.4       59.0865       10855.2 -1.000000E+10 -1.000000E+10 -1.000000E+10 -1.000000E+10 -1.000000E+10  0.000000E+00  0.000000E+00  0.000000E+00  0.000000E+00  0.000000E+00  0.000000E+00  0.000000E+00  0.000000E+00  0.000000E+00  0.000000E+00  0.000000E+00  0.000000E+00       Funtag_D
      65  4.935198E+05  4.514103E+05       42109.5       7.45735      0.118619       1.58075      0.211768      0.231699     0.0441711     0.0193728    0.00786516       1.10288       Opttag_D
      65       46.0833       2.24995       1.00000  0.000000E+00       1.00000  0.000000E+00      0.796581       65.8542       1.00000       24.6001  0.000000E+00       1.01560  0.000000E+00       1.01560  0.000000E+00  0.000000E+00      -2.03121       45.1306       1.58302        1       Scatag_D
      65       1744.09       6638.52  0.000000E+00  0.000000E+00       2202.63  0.000000E+00       3678.64       5808.36  0.000000E+00       11124.4       58.9894       10853.9 -1.000000E+10 -1.000000E+10 -1.000000E+10 -1.000000E+10 -1.000000E+10  0.000000E+00  0.000000E+00  0.000000E+00  0.000000E+00  0.000000E+00  0.000000E+00  0.000000E+00  0.000000E+00  0.000000E+00  0.000000E+00  0.000000E+00  0.000000E+00       Funtag_D
      66  4.934714E+05  4.513859E+05       42085.6       9.90697      0.119502       1.58338      0.211764      0.231695     0.0442312     0.0194956    0.00787145       1.10230       Opttag_D
      66       46.0856       2.24871       1.00000  0.000000E+00       1.00000  0.000000E+00      0.796487       65.8423       1.00000       24.6145  0.000000E+00       1.01692  0.000000E+00       1.01692  0.000000E+00  0.000000E+00      -2.03384       45.1306       1.58302        1       Scatag_D
      66       1746.88       6631.57  0.000000E+00  0.000000E+00       2203.05  0.000000E+00       3685.86       5796.49  0.000000E+00       11115.9       58.8618       10846.9 -1.000000E+10 -1.000000E+10 -1.000000E+10 -1.000000E+10 -1.000000E+10  0.000000E+00  0.000000E+00  0.000000E+00  0.000000E+00  0.000000E+00  0.000000E+00  0.000000E+00  0.000000E+00  0.000000E+00  0.000000E+00  0.000000E+00  0.000000E+00       Funtag_D
      67  4.934641E+05  4.513604E+05       42103.7       23.9773      0.122267       1.60369      0.211786      0.231765     0.0442938     0.0194760    0.00788890       1.10194       Opttag_D
      67       46.0972       2.24734       1.00000  0.000000E+00       1.00000  0.000000E+00      0.796272       65.8114       1.00000       24.6650  0.000000E+00       1.01929  0.000000E+00       1.01929  0.000000E+00  0.000000E+00      -2.03857       45.1306       1.58302        1       Scatag_D
      67       1754.63       6627.27  0.000000E+00  0.000000E+00       2205.04  0.000000E+00       3696.15       5817.02  0.000000E+00       11107.5       59.0136       10837.0 -1.000000E+10 -1.000000E+10 -1.000000E+10 -1.000000E+10 -1.000000E+10  0.000000E+00  0.000000E+00  0.000000E+00  0.000000E+00  0.000000E+00  0.000000E+00  0.000000E+00  0.000000E+00  0.000000E+00  0.000000E+00  0.000000E+00  0.000000E+00       Funtag_D
      68  4.934162E+05  4.513596E+05       42056.6       9.05441      0.122186       1.60339      0.211784      0.231764     0.0442958     0.0194826    0.00788004       1.10175       Opttag_D
      68       46.0969       2.24730       1.00000  0.000000E+00       1.00000  0.000000E+00      0.796279       65.8120       1.00000       24.6643  0.000000E+00       1.01919  0.000000E+00       1.01919  0.000000E+00  0.000000E+00      -2.03837       45.1306       1.58302        1       Scatag_D
      68       1750.69       6624.92  0.000000E+00  0.000000E+00       2204.96  0.000000E+00       3694.56       5777.90  0.000000E+00       11108.1       59.0283       10836.4 -1.000000E+10 -1.000000E+10 -1.000000E+10 -1.000000E+10 -1.000000E+10  0.000000E+00  0.000000E+00  0.000000E+00  0.000000E+00  0.000000E+00  0.000000E+00  0.000000E+00  0.000000E+00  0.000000E+00  0.000000E+00  0.000000E+00  0.000000E+00       Funtag_D
      69  4.933942E+05  4.513539E+05       42040.3       6.29337      0.123151       1.61130      0.211795      0.231796     0.0443099     0.0194484    0.00787685       1.10165       Opttag_D
      69       46.1014       2.24694       1.00000  0.000000E+00       1.00000  0.000000E+00      0.796211       65.8010       1.00000       24.6834  0.000000E+00       1.01982  0.000000E+00       1.01982  0.000000E+00  0.000000E+00      -2.03965       45.1306       1.58302        1       Scatag_D
      69       1749.28       6623.73  0.000000E+00  0.000000E+00       2205.74  0.000000E+00       3695.61       5767.21  0.000000E+00       11107.1       59.1463       10832.5 -1.000000E+10 -1.000000E+10 -1.000000E+10 -1.000000E+10 -1.000000E+10  0.000000E+00  0.000000E+00  0.000000E+00  0.000000E+00  0.000000E+00  0.000000E+00  0.000000E+00  0.000000E+00  0.000000E+00  0.000000E+00  0.000000E+00  0.000000E+00       Funtag_D
      70  4.933674E+05  4.513431E+05       42024.3       7.65507      0.125098       1.62499      0.211817      0.231863     0.0443364     0.0193751    0.00787534       1.10150       Opttag_D
      70       46.1099       2.24621       1.00000  0.000000E+00       1.00000  0.000000E+00      0.796074       65.7787       1.00000       24.7213  0.000000E+00       1.02125  0.000000E+00       1.02125  0.000000E+00  0.000000E+00      -2.04249       45.1306       1.58302        1       Scatag_D
      70       1748.60       6621.88  0.000000E+00  0.000000E+00       2207.42  0.000000E+00       3698.91       5758.36  0.000000E+00       11104.8       59.3543       10825.0 -1.000000E+10 -1.000000E+10 -1.000000E+10 -1.000000E+10 -1.000000E+10  0.000000E+00  0.000000E+00  0.000000E+00  0.000000E+00  0.000000E+00  0.000000E+00  0.000000E+00  0.000000E+00  0.000000E+00  0.000000E+00  0.000000E+00  0.000000E+00       Funtag_D
      71  4.933354E+05  4.513242E+05       42011.2       12.1348      0.127567       1.63795      0.211845      0.231957     0.0443828     0.0192862    0.00786783       1.10148       Opttag_D
      71       46.1200       2.24494       1.00000  0.000000E+00       1.00000  0.000000E+00      0.795899       65.7473       1.00000       24.7700  0.000000E+00       1.02327  0.000000E+00       1.02327  0.000000E+00  0.000000E+00      -2.04655       45.1306       1.58302        1       Scatag_D
      71       1745.27       6621.69  0.000000E+00  0.000000E+00       2209.68  0.000000E+00       3703.38       5755.42  0.000000E+00       11101.3       59.5439       10814.9 -1.000000E+10 -1.000000E+10 -1.000000E+10 -1.000000E+10 -1.000000E+10  0.000000E+00  0.000000E+00  0.000000E+00  0.000000E+00  0.000000E+00  0.000000E+00  0.000000E+00  0.000000E+00  0.000000E+00  0.000000E+00  0.000000E+00  0.000000E+00       Funtag_D
      72  4.932981E+05  4.513053E+05       41992.8       7.45980      0.130369       1.64914      0.211880      0.232073     0.0444294     0.0191487    0.00786267       1.10142       Opttag_D
      72       46.1311       2.24364       1.00000  0.000000E+00       1.00000  0.000000E+00      0.795704       65.7132       1.00000       24.8249  0.000000E+00       1.02541  0.000000E+00       1.02541  0.000000E+00  0.000000E+00      -2.05081       45.1306       1.58302        1       Scatag_D
      72       1742.98       6621.02  0.000000E+00  0.000000E+00       2212.52  0.000000E+00       3708.09       5745.58  0.000000E+00       11098.4       59.7049       10804.6 -1.000000E+10 -1.000000E+10 -1.000000E+10 -1.000000E+10 -1.000000E+10  0.000000E+00  0.000000E+00  0.000000E+00  0.000000E+00  0.000000E+00  0.000000E+00  0.000000E+00  0.000000E+00  0.000000E+00  0.000000E+00  0.000000E+00  0.000000E+00       Funtag_D
      73  4.932694E+05  4.512860E+05       41983.5       6.73984      0.130523       1.64379      0.211871      0.232076     0.0444770     0.0191594    0.00785523       1.10128       Opttag_D
      73       46.1309       2.24287       1.00000  0.000000E+00       1.00000  0.000000E+00      0.795679       65.7100       1.00000       24.8269  0.000000E+00       1.02554  0.000000E+00       1.02554  0.000000E+00  0.000000E+00      -2.05107       45.1306       1.58302        1       Scatag_D
      73       1739.68       6619.28  0.000000E+00  0.000000E+00       2212.59  0.000000E+00       3708.97       5745.63  0.000000E+00       11095.5       59.5622       10802.2 -1.000000E+10 -1.000000E+10 -1.000000E+10 -1.000000E+10 -1.000000E+10  0.000000E+00  0.000000E+00  0.000000E+00  0.000000E+00  0.000000E+00  0.000000E+00  0.000000E+00  0.000000E+00  0.000000E+00  0.000000E+00  0.000000E+00  0.000000E+00       Funtag_D
      74  4.932224E+05  4.512395E+05       41982.9       10.7595      0.130649       1.62678      0.211842      0.232066     0.0445912     0.0192151    0.00784666       1.10105       Opttag_D
      74       46.1294       2.24111       1.00000  0.000000E+00       1.00000  0.000000E+00      0.795633       65.7077       1.00000       24.8252  0.000000E+00       1.02547  0.000000E+00       1.02547  0.000000E+00  0.000000E+00      -2.05094       45.1306       1.58302        1       Scatag_D
      74       1735.89       6616.49  0.000000E+00  0.000000E+00       2212.44  0.000000E+00       3711.57       5760.86  0.000000E+00       11088.7       59.1475       10797.9 -1.000000E+10 -1.000000E+10 -1.000000E+10 -1.000000E+10 -1.000000E+10  0.000000E+00  0.000000E+00  0.000000E+00  0.000000E+00  0.000000E+00  0.000000E+00  0.000000E+00  0.000000E+00  0.000000E+00  0.000000E+00  0.000000E+00  0.000000E+00       Funtag_D
      75  4.932040E+05  4.512023E+05       42001.8       19.4163      0.131774       1.61115      0.211834      0.232073     0.0446828     0.0192584    0.00784952       1.10105       Opttag_D
      75       46.1324       2.23956       1.00000  0.000000E+00       1.00000  0.000000E+00      0.795534       65.6978       1.00000       24.8410  0.000000E+00       1.02621  0.000000E+00       1.02621  0.000000E+00  0.000000E+00      -2.05241       45.1306       1.58302        1       Scatag_D
      75       1737.16       6616.50  0.000000E+00  0.000000E+00       2213.31  0.000000E+00       3714.84       5786.87  0.000000E+00       11083.0       58.7868       10791.4 -1.000000E+10 -1.000000E+10 -1.000000E+10 -1.000000E+10 -1.000000E+10  0.000000E+00  0.000000E+00  0.000000E+00  0.000000E+00  0.000000E+00  0.000000E+00  0.000000E+00  0.000000E+00  0.000000E+00  0.000000E+00  0.000000E+00  0.000000E+00       Funtag_D
      76  4.931657E+05  4.512022E+05       41963.4       8.79769      0.131518       1.60897      0.211830      0.232060     0.0446830     0.0192675    0.00784833       1.10050       Opttag_D
      76       46.1315       2.23967       1.00000  0.000000E+00       1.00000  0.000000E+00      0.795550       65.7026       1.00000       24.8353  0.000000E+00       1.02578  0.000000E+00       1.02578  0.000000E+00  0.000000E+00      -2.05156       45.1306       1.58302        1       Scatag_D
      76       1736.63       6609.95  0.000000E+00  0.000000E+00       2213.00  0.000000E+00       3713.56       5758.77  0.000000E+00       11082.7       58.7334       10790.1 -1.000000E+10 -1.000000E+10 -1.000000E+10 -1.000000E+10 -1.000000E+10  0.000000E+00  0.000000E+00  0.000000E+00  0.000000E+00  0.000000E+00  0.000000E+00  0.000000E+00  0.000000E+00  0.000000E+00  0.000000E+00  0.000000E+00  0.000000E+00       Funtag_D
      77  4.931353E+05  4.511989E+05       41936.3       6.63185      0.131758       1.60396      0.211830      0.232050     0.0446909     0.0192756    0.00785115       1.09994       Opttag_D
      77       46.1327       2.23964       1.00000  0.000000E+00       1.00000  0.000000E+00      0.795533       65.7045       1.00000       24.8373  0.000000E+00       1.02554  0.000000E+00       1.02554  0.000000E+00  0.000000E+00      -2.05108       45.1306       1.58302        1       Scatag_D
      77       1737.88       6603.16  0.000000E+00  0.000000E+00       2213.13  0.000000E+00       3712.06       5744.84  0.000000E+00       11081.2       58.6178       10785.5 -1.000000E+10 -1.000000E+10 -1.000000E+10 -1.000000E+10 -1.000000E+10  0.000000E+00  0.000000E+00  0.000000E+00  0.000000E+00  0.000000E+00  0.000000E+00  0.000000E+00  0.000000E+00  0.000000E+00  0.000000E+00  0.000000E+00  0.000000E+00       Funtag_D
      78  4.931094E+05  4.511958E+05       41913.5       7.79932      0.132378       1.59582      0.211834      0.232045     0.0446986     0.0192833    0.00785629       1.09932       Opttag_D
      78       46.1358       2.23964       1.00000  0.000000E+00       1.00000  0.000000E+00      0.795492       65.7051       1.00000       24.8447  0.000000E+00       1.02547  0.000000E+00       1.02547  0.000000E+00  0.000000E+00      -2.05095       45.1306       1.58302        1       Scatag_D
      78       1740.16       6595.74  0.000000E+00  0.000000E+00       2213.65  0.000000E+00       3710.97       5737.57  0.000000E+00       11078.2       58.4722       10778.8 -1.000000E+10 -1.000000E+10 -1.000000E+10 -1.000000E+10 -1.000000E+10  0.000000E+00  0.000000E+00  0.000000E+00  0.000000E+00  0.000000E+00  0.000000E+00  0.000000E+00  0.000000E+00  0.000000E+00  0.000000E+00  0.000000E+00  0.000000E+00       Funtag_D
      79  4.930665E+05  4.512023E+05       41864.2       10.4511      0.133435       1.58092      0.211846      0.232039     0.0446828     0.0193146    0.00784716       1.09834       Opttag_D
      79       46.1432       2.24040       1.00000  0.000000E+00       1.00000  0.000000E+00      0.795431       65.7101       1.00000       24.8572  0.000000E+00       1.02511  0.000000E+00       1.02511  0.000000E+00  0.000000E+00      -2.05021       45.1306       1.58302        1       Scatag_D
      79       1736.11       6584.05  0.000000E+00  0.000000E+00       2214.72  0.000000E+00       3709.51       5724.64  0.000000E+00       11070.2       58.3139       10766.7 -1.000000E+10 -1.000000E+10 -1.000000E+10 -1.000000E+10 -1.000000E+10  0.000000E+00  0.000000E+00  0.000000E+00  0.000000E+00  0.000000E+00  0.000000E+00  0.000000E+00  0.000000E+00  0.000000E+00  0.000000E+00  0.000000E+00  0.000000E+00       Funtag_D
      80  4.930399E+05  4.512115E+05       41828.3       11.4618      0.134441       1.56686      0.211852      0.232038     0.0446600     0.0193505    0.00783911       1.09787       Opttag_D
      80       46.1507       2.24130       1.00000  0.000000E+00       1.00000  0.000000E+00      0.795383       65.7149       1.00000       24.8689  0.000000E+00       1.02474  0.000000E+00       1.02474  0.000000E+00  0.000000E+00      -2.04949       45.1306       1.58302        2       Scatag_D
      80       1732.55       6578.39  0.000000E+00  0.000000E+00       2215.79  0.000000E+00       3707.94       5715.26  0.000000E+00       11062.9       58.2191       10757.3 -1.000000E+10 -1.000000E+10 -1.000000E+10 -1.000000E+10 -1.000000E+10  0.000000E+00  0.000000E+00  0.000000E+00  0.000000E+00  0.000000E+00  0.000000E+00  0.000000E+00  0.000000E+00  0.000000E+00  0.000000E+00  0.000000E+00  0.000000E+00       Funtag_D
      81  4.930113E+05  4.512174E+05       41793.8       6.87589      0.135077       1.55817      0.211858      0.232044     0.0446455     0.0193728    0.00782400       1.09750       Opttag_D
      81       46.1563       2.24201       1.00000  0.000000E+00       1.00000  0.000000E+00      0.795356       65.7187       1.00000       24.8769  0.000000E+00       1.02433  0.000000E+00       1.02433  0.000000E+00  0.000000E+00      -2.04865       45.1306       1.58302        1       Scatag_D
      81       1725.88       6573.89  0.000000E+00  0.000000E+00       2216.58  0.000000E+00       3706.60       5704.73  0.000000E+00       11056.9       58.2029       10751.0 -1.000000E+10 -1.000000E+10 -1.000000E+10 -1.000000E+10 -1.000000E+10  0.000000E+00  0.000000E+00  0.000000E+00  0.000000E+00  0.000000E+00  0.000000E+00  0.000000E+00  0.000000E+00  0.000000E+00  0.000000E+00  0.000000E+00  0.000000E+00       Funtag_D
      82  4.929835E+05  4.512146E+05       41768.8       7.08057      0.135702       1.54936      0.211851      0.232056     0.0446524     0.0194121    0.00780444       1.09744       Opttag_D
      82       46.1622       2.24251       1.00000  0.000000E+00       1.00000  0.000000E+00      0.795333       65.7223       1.00000       24.8852  0.000000E+00       1.02354  0.000000E+00       1.02354  0.000000E+00  0.000000E+00      -2.04707       45.1306       1.58302        1       Scatag_D
      82       1717.26       6573.22  0.000000E+00  0.000000E+00       2217.37  0.000000E+00       3704.30       5703.94  0.000000E+00       11049.5       58.1715       10745.0 -1.000000E+10 -1.000000E+10 -1.000000E+10 -1.000000E+10 -1.000000E+10  0.000000E+00  0.000000E+00  0.000000E+00  0.000000E+00  0.000000E+00  0.000000E+00  0.000000E+00  0.000000E+00  0.000000E+00  0.000000E+00  0.000000E+00  0.000000E+00       Funtag_D
      83  4.929515E+05  4.512035E+05       41747.9       10.5337      0.136798       1.53443      0.211842      0.232084     0.0446797     0.0194633    0.00778156       1.09758       Opttag_D
      83       46.1719       2.24307       1.00000  0.000000E+00       1.00000  0.000000E+00      0.795296       65.7266       1.00000       24.9000  0.000000E+00       1.02194  0.000000E+00       1.02194  0.000000E+00  0.000000E+00      -2.04389       45.1306       1.58302        1       Scatag_D
      83       1707.21       6574.92  0.000000E+00  0.000000E+00       2218.70  0.000000E+00       3699.62       5715.04  0.000000E+00       11038.1       58.0266       10736.3 -1.000000E+10 -1.000000E+10 -1.000000E+10 -1.000000E+10 -1.000000E+10  0.000000E+00  0.000000E+00  0.000000E+00  0.000000E+00  0.000000E+00  0.000000E+00  0.000000E+00  0.000000E+00  0.000000E+00  0.000000E+00  0.000000E+00  0.000000E+00       Funtag_D
      84  4.929213E+05  4.511818E+05       41739.5       10.7147      0.137800       1.52112      0.211827      0.232114     0.0447332     0.0195289    0.00776552       1.09782       Opttag_D
      84       46.1803       2.24317       1.00000  0.000000E+00       1.00000  0.000000E+00      0.795264       65.7286       1.00000       24.9148  0.000000E+00       1.01994  0.000000E+00       1.01994  0.000000E+00  0.000000E+00      -2.03987       45.1306       1.58302        1       Scatag_D
      84       1700.17       6577.76  0.000000E+00  0.000000E+00       2219.86  0.000000E+00       3694.52       5732.23  0.000000E+00       11028.0       57.8006       10729.2 -1.000000E+10 -1.000000E+10 -1.000000E+10 -1.000000E+10 -1.000000E+10  0.000000E+00  0.000000E+00  0.000000E+00  0.000000E+00  0.000000E+00  0.000000E+00  0.000000E+00  0.000000E+00  0.000000E+00  0.000000E+00  0.000000E+00  0.000000E+00       Funtag_D
      85  4.928973E+05  4.511664E+05       41730.9       7.07006      0.138012       1.51837      0.211818      0.232121     0.0447711     0.0195571    0.00776385       1.09756       Opttag_D
      85       46.1816       2.24274       1.00000  0.000000E+00       1.00000  0.000000E+00      0.795256       65.7266       1.00000       24.9184  0.000000E+00       1.01904  0.000000E+00       1.01904  0.000000E+00  0.000000E+00      -2.03808       45.1306       1.58302        1       Scatag_D
      85       1699.44       6574.66  0.000000E+00  0.000000E+00       2220.00  0.000000E+00       3692.92       5734.66  0.000000E+00       11025.0       57.6539       10726.5 -1.000000E+10 -1.000000E+10 -1.000000E+10 -1.000000E+10 -1.000000E+10  0.000000E+00  0.000000E+00  0.000000E+00  0.000000E+00  0.000000E+00  0.000000E+00  0.000000E+00  0.000000E+00  0.000000E+00  0.000000E+00  0.000000E+00  0.000000E+00       Funtag_D
      86  4.928704E+05  4.511423E+05       41728.1       6.81544      0.138381       1.51402      0.211810      0.232135     0.0448304     0.0196037    0.00776358       1.09717       Opttag_D
      86       46.1834       2.24201       1.00000  0.000000E+00       1.00000  0.000000E+00      0.795240       65.7221       1.00000       24.9252  0.000000E+00       1.01748  0.000000E+00       1.01748  0.000000E+00  0.000000E+00      -2.03496       45.1306       1.58302        1       Scatag_D
      86       1699.33       6569.95  0.000000E+00  0.000000E+00       2220.23  0.000000E+00       3690.94       5749.00  0.000000E+00       11020.0       57.4051       10721.2 -1.000000E+10 -1.000000E+10 -1.000000E+10 -1.000000E+10 -1.000000E+10  0.000000E+00  0.000000E+00  0.000000E+00  0.000000E+00  0.000000E+00  0.000000E+00  0.000000E+00  0.000000E+00  0.000000E+00  0.000000E+00  0.000000E+00  0.000000E+00       Funtag_D
      87  4.928478E+05  4.511300E+05       41717.8       8.28384      0.138962       1.50770      0.211808      0.232153     0.0448606     0.0196738    0.00775431       1.09686       Opttag_D
      87       46.1873       2.24185       1.00000  0.000000E+00       1.00000  0.000000E+00      0.795223       65.7182       1.00000       24.9346  0.000000E+00       1.01579  0.000000E+00       1.01579  0.000000E+00  0.000000E+00      -2.03157       45.1306       1.58302        1       Scatag_D
      87       1695.27       6566.26  0.000000E+00  0.000000E+00       2220.79  0.000000E+00       3689.38       5762.02  0.000000E+00       11013.1       57.2397       10713.7 -1.000000E+10 -1.000000E+10 -1.000000E+10 -1.000000E+10 -1.000000E+10  0.000000E+00  0.000000E+00  0.000000E+00  0.000000E+00  0.000000E+00  0.000000E+00  0.000000E+00  0.000000E+00  0.000000E+00  0.000000E+00  0.000000E+00  0.000000E+00       Funtag_D
      88  4.928129E+05  4.511240E+05       41688.9       8.27628      0.140218       1.49500      0.211817      0.232193     0.0448754     0.0197955    0.00773361       1.09640       Opttag_D
      88       46.1960       2.24228       1.00000  0.000000E+00       1.00000  0.000000E+00      0.795193       65.7123       1.00000       24.9544  0.000000E+00       1.01277  0.000000E+00       1.01277  0.000000E+00  0.000000E+00      -2.02554       45.1306       1.58302        1       Scatag_D
      88       1686.23       6560.72  0.000000E+00  0.000000E+00       2222.25  0.000000E+00       3686.77       5778.38  0.000000E+00       10999.6       57.0621       10697.9 -1.000000E+10 -1.000000E+10 -1.000000E+10 -1.000000E+10 -1.000000E+10  0.000000E+00  0.000000E+00  0.000000E+00  0.000000E+00  0.000000E+00  0.000000E+00  0.000000E+00  0.000000E+00  0.000000E+00  0.000000E+00  0.000000E+00  0.000000E+00       Funtag_D
      89  4.927881E+05  4.511418E+05       41646.3       12.7116      0.141663       1.48173      0.211842      0.232230     0.0448314     0.0199347    0.00770512       1.09656       Opttag_D
      89       46.2057       2.24357       1.00000  0.000000E+00       1.00000  0.000000E+00      0.795175       65.7079       1.00000       24.9753  0.000000E+00       1.01032  0.000000E+00       1.01032  0.000000E+00  0.000000E+00      -2.02065       45.1306       1.58302        1       Scatag_D
      89       1673.83       6562.63  0.000000E+00  0.000000E+00       2224.06  0.000000E+00       3683.51       5782.87  0.000000E+00       10983.6       57.1166       10678.6 -1.000000E+10 -1.000000E+10 -1.000000E+10 -1.000000E+10 -1.000000E+10  0.000000E+00  0.000000E+00  0.000000E+00  0.000000E+00  0.000000E+00  0.000000E+00  0.000000E+00  0.000000E+00  0.000000E+00  0.000000E+00  0.000000E+00  0.000000E+00       Funtag_D
      90  4.927613E+05  4.511555E+05       41605.8       6.83381      0.142155       1.47773      0.211851      0.232244     0.0447978     0.0199691    0.00769231       1.09633       Opttag_D
      90       46.2088       2.24425       1.00000  0.000000E+00       1.00000  0.000000E+00      0.795173       65.7069       1.00000       24.9817  0.000000E+00       1.00998  0.000000E+00       1.00998  0.000000E+00  0.000000E+00      -2.01996       45.1306       1.58302        1       Scatag_D
      90       1668.27       6559.95  0.000000E+00  0.000000E+00       2224.75  0.000000E+00       3682.40       5765.30  0.000000E+00       10977.0       57.2525       10670.9 -1.000000E+10 -1.000000E+10 -1.000000E+10 -1.000000E+10 -1.000000E+10  0.000000E+00  0.000000E+00  0.000000E+00  0.000000E+00  0.000000E+00  0.000000E+00  0.000000E+00  0.000000E+00  0.000000E+00  0.000000E+00  0.000000E+00  0.000000E+00       Funtag_D
      91  4.927384E+05  4.511724E+05       41566.0       5.95630      0.142709       1.47373      0.211865      0.232261     0.0447564     0.0200054    0.00767984       1.09633       Opttag_D
      91       46.2116       2.24501       1.00000  0.000000E+00       1.00000  0.000000E+00      0.795174       65.7058       1.00000       24.9880  0.000000E+00       1.00988  0.000000E+00       1.00988  0.000000E+00  0.000000E+00      -2.01977       45.1306       1.58302        1       Scatag_D
      91       1662.86       6559.93  0.000000E+00  0.000000E+00       2225.44  0.000000E+00       3681.46       5748.84  0.000000E+00       10967.9       57.4781       10662.1 -1.000000E+10 -1.000000E+10 -1.000000E+10 -1.000000E+10 -1.000000E+10  0.000000E+00  0.000000E+00  0.000000E+00  0.000000E+00  0.000000E+00  0.000000E+00  0.000000E+00  0.000000E+00  0.000000E+00  0.000000E+00  0.000000E+00  0.000000E+00       Funtag_D
      92  4.927152E+05  4.511885E+05       41526.7       8.43739      0.143623       1.46750      0.211884      0.232283     0.0447167     0.0200580    0.00767079       1.09655       Opttag_D
      92       46.2151       2.24564       1.00000  0.000000E+00       1.00000  0.000000E+00      0.795167       65.7011       1.00000       24.9999  0.000000E+00       1.00978  0.000000E+00       1.00978  0.000000E+00  0.000000E+00      -2.01957       45.1306       1.58302        1       Scatag_D
      92       1658.95       6562.56  0.000000E+00  0.000000E+00       2226.42  0.000000E+00       3680.58       5735.86  0.000000E+00       10954.1       57.6869       10650.6 -1.000000E+10 -1.000000E+10 -1.000000E+10 -1.000000E+10 -1.000000E+10  0.000000E+00  0.000000E+00  0.000000E+00  0.000000E+00  0.000000E+00  0.000000E+00  0.000000E+00  0.000000E+00  0.000000E+00  0.000000E+00  0.000000E+00  0.000000E+00       Funtag_D
      93  4.926879E+05  4.512071E+05       41480.9       10.2089      0.145357       1.45666      0.211921      0.232327     0.0446710     0.0201164    0.00766069       1.09709       Opttag_D
      93       46.2204       2.24620       1.00000  0.000000E+00       1.00000  0.000000E+00      0.795148       65.6876       1.00000       25.0250  0.000000E+00       1.00937  0.000000E+00       1.00937  0.000000E+00  0.000000E+00      -2.01873       45.1306       1.58302        1       Scatag_D
      93       1654.58       6569.05  0.000000E+00  0.000000E+00       2228.19  0.000000E+00       3679.85       5727.89  0.000000E+00       10930.6       57.9219       10632.8 -1.000000E+10 -1.000000E+10 -1.000000E+10 -1.000000E+10 -1.000000E+10  0.000000E+00  0.000000E+00  0.000000E+00  0.000000E+00  0.000000E+00  0.000000E+00  0.000000E+00  0.000000E+00  0.000000E+00  0.000000E+00  0.000000E+00  0.000000E+00       Funtag_D
      94  4.926626E+05  4.511980E+05       41464.6       7.35133      0.146018       1.45374      0.211931      0.232340     0.0446933     0.0201219    0.00766120       1.09727       Opttag_D
      94       46.2204       2.24548       1.00000  0.000000E+00       1.00000  0.000000E+00      0.795128       65.6764       1.00000       25.0373  0.000000E+00       1.00955  0.000000E+00       1.00955  0.000000E+00  0.000000E+00      -2.01911       45.1306       1.58302        1       Scatag_D
      94       1654.80       6571.22  0.000000E+00  0.000000E+00       2228.61  0.000000E+00       3681.25       5723.50  0.000000E+00       10920.2       57.8618       10627.2 -1.000000E+10 -1.000000E+10 -1.000000E+10 -1.000000E+10 -1.000000E+10  0.000000E+00  0.000000E+00  0.000000E+00  0.000000E+00  0.000000E+00  0.000000E+00  0.000000E+00  0.000000E+00  0.000000E+00  0.000000E+00  0.000000E+00  0.000000E+00       Funtag_D
      95  4.926351E+05  4.511739E+05       41461.2       6.94387      0.146793       1.45136      0.211936      0.232356     0.0447525     0.0201043    0.00766273       1.09753       Opttag_D
      95       46.2189       2.24387       1.00000  0.000000E+00       1.00000  0.000000E+00      0.795093       65.6574       1.00000       25.0546  0.000000E+00       1.00992  0.000000E+00       1.00992  0.000000E+00  0.000000E+00      -2.01985       45.1306       1.58302        1       Scatag_D
      95       1655.47       6574.29  0.000000E+00  0.000000E+00       2228.90  0.000000E+00       3684.11       5731.58  0.000000E+00       10907.4       57.6737       10621.8 -1.000000E+10 -1.000000E+10 -1.000000E+10 -1.000000E+10 -1.000000E+10  0.000000E+00  0.000000E+00  0.000000E+00  0.000000E+00  0.000000E+00  0.000000E+00  0.000000E+00  0.000000E+00  0.000000E+00  0.000000E+00  0.000000E+00  0.000000E+00       Funtag_D
      96  4.926101E+05  4.511494E+05       41460.7       8.53531      0.147829       1.44900      0.211948      0.232395     0.0448129     0.0200408    0.00766413       1.09790       Opttag_D
      96       46.2173       2.24202       1.00000  0.000000E+00       1.00000  0.000000E+00      0.795047       65.6320       1.00000       25.0781  0.000000E+00       1.01039  0.000000E+00       1.01039  0.000000E+00  0.000000E+00      -2.02079       45.1306       1.58302        1       Scatag_D
      96       1656.07       6578.72  0.000000E+00  0.000000E+00       2229.50  0.000000E+00       3687.30       5744.84  0.000000E+00       10891.4       57.5005       10615.3 -1.000000E+10 -1.000000E+10 -1.000000E+10 -1.000000E+10 -1.000000E+10  0.000000E+00  0.000000E+00  0.000000E+00  0.000000E+00  0.000000E+00  0.000000E+00  0.000000E+00  0.000000E+00  0.000000E+00  0.000000E+00  0.000000E+00  0.000000E+00       Funtag_D
      97  4.925839E+05  4.511199E+05       41463.9       10.3882      0.149488       1.44675      0.211976      0.232470     0.0448853     0.0199246    0.00766628       1.09843       Opttag_D
      97       46.2142       2.23929       1.00000  0.000000E+00       1.00000  0.000000E+00      0.794981       65.5890       1.00000       25.1162  0.000000E+00       1.01149  0.000000E+00       1.01149  0.000000E+00  0.000000E+00      -2.02299       45.1306       1.58302        1       Scatag_D
      97       1657.00       6585.05  0.000000E+00  0.000000E+00       2230.53  0.000000E+00       3691.50       5768.73  0.000000E+00       10867.8       57.2915       10606.1 -1.000000E+10 -1.000000E+10 -1.000000E+10 -1.000000E+10 -1.000000E+10  0.000000E+00  0.000000E+00  0.000000E+00  0.000000E+00  0.000000E+00  0.000000E+00  0.000000E+00  0.000000E+00  0.000000E+00  0.000000E+00  0.000000E+00  0.000000E+00       Funtag_D
      98  4.925595E+05  4.511216E+05       41437.9       6.82343      0.149992       1.44747      0.211989      0.232511     0.0448813     0.0198517    0.00765783       1.09826       Opttag_D
      98       46.2127       2.23872       1.00000  0.000000E+00       1.00000  0.000000E+00      0.794962       65.5750       1.00000       25.1273  0.000000E+00       1.01229  0.000000E+00       1.01229  0.000000E+00  0.000000E+00      -2.02459       45.1306       1.58302        1       Scatag_D
      98       1653.35       6582.99  0.000000E+00  0.000000E+00       2230.94  0.000000E+00       3691.26       5761.88  0.000000E+00       10859.9       57.2755       10600.3 -1.000000E+10 -1.000000E+10 -1.000000E+10 -1.000000E+10 -1.000000E+10  0.000000E+00  0.000000E+00  0.000000E+00  0.000000E+00  0.000000E+00  0.000000E+00  0.000000E+00  0.000000E+00  0.000000E+00  0.000000E+00  0.000000E+00  0.000000E+00       Funtag_D
      99  4.925374E+05  4.511350E+05       41402.4       6.34382      0.150540       1.44898      0.212009      0.232556     0.0448483     0.0197669    0.00764775       1.09794       Opttag_D
      99       46.2109       2.23851       1.00000  0.000000E+00       1.00000  0.000000E+00      0.794944       65.5614       1.00000       25.1373  0.000000E+00       1.01343  0.000000E+00       1.01343  0.000000E+00  0.000000E+00      -2.02685       45.1306       1.58302        1       Scatag_D
      99       1649.00       6579.19  0.000000E+00  0.000000E+00       2231.47  0.000000E+00       3689.87       5752.67  0.000000E+00       10850.9       57.2992       10592.0 -1.000000E+10 -1.000000E+10 -1.000000E+10 -1.000000E+10 -1.000000E+10  0.000000E+00  0.000000E+00  0.000000E+00  0.000000E+00  0.000000E+00  0.000000E+00  0.000000E+00  0.000000E+00  0.000000E+00  0.000000E+00  0.000000E+00  0.000000E+00       Funtag_D

   MaxLik Scaling Cycle 0106

   Wilson plot

   Statistics in resolution bins after refinement cycle 0003
```


|  |  |
| --- | --- |
|  | - Rxpct factors plot- Rxpct factors table- <Log-Lik Gain>- Ln Sigma A- Correlation Coefficients |


```
   Iters     Tot_Funct    Xray_Funct    Geom_Funct          Grms     rmsD2init     maxD2init             R         Rfree           LLG       LLGfree     rms(bond)    rms(angle)
   Iters             K   B_IMPF_FRAG        K_MISS   B_TEMP_MISS   K_IMPF_MISS   B_IMPF_MISS        K_SOLV   B_TEMP_SOLV   K_IMPF_SOLV   B_IMPF_SOLV        B_TEMP     B_TEMP_11     B_TEMP_12     B_TEMP_22     B_TEMP_13     B_TEMP_23     B_TEMP_33      K_WILSON      B_WILSON      FGCYCLES
   Iters          BOND         ANGLE      IMPROPER       TORSION    SINTORSION      TRIGONAL        PLANAR  BCORRELATION       CONTACT     IDEALDIST        CHIRAL    SIMILARITY       OCCSSUM  UTILDISTANCE     UTILANGLE   UTILTORSION        RHOFIT        ADP-01        ADP-02        ADP-03        ADP-04        ADP-05        ADP-06        ADP-07        ADP-08        ADP-09        ADP-10      RHOFITPC            QM
     100  4.925139E+05  4.511593E+05       41354.6       8.45606      0.151656       1.45561      0.212050      0.232618     0.0447885     0.0196582    0.00763349       1.09777       Opttag_D
     100       46.2072       2.23799       1.00000  0.000000E+00       1.00000  0.000000E+00      0.794910       65.5343       1.00000       25.1568  0.000000E+00       1.01568  0.000000E+00       1.01568  0.000000E+00  0.000000E+00      -2.03136       45.1306       1.58302        1       Scatag_D
     100       1642.85       6577.11  0.000000E+00  0.000000E+00       2232.49  0.000000E+00       3688.63       5743.48  0.000000E+00       10834.7       57.2675       10578.1 -1.000000E+10 -1.000000E+10 -1.000000E+10 -1.000000E+10 -1.000000E+10  0.000000E+00  0.000000E+00  0.000000E+00  0.000000E+00  0.000000E+00  0.000000E+00  0.000000E+00  0.000000E+00  0.000000E+00  0.000000E+00  0.000000E+00  0.000000E+00       Funtag_D
   Geometry Restraint Deviations SCREEN Information

 
 No -bcorrel_zeroocchydrogens_factor flag found - keeping default: will set B for OCC=0 hydrogens to    1.200 times heavy-atom B


 TLS information. Total number of groups:     2


 Group   Natoms    FixRB   FixTLS   Group Tag    Group Spec
     1     2785      Y        Y     tls1    { A|* }
     2     2772      Y        Y     tls2    { B|* }
 
NOTE BUSTER_TLS_SET tls1 { A|* }
NOTE BUSTER_TLS_T   tls1     -1.42739E-01 -2.31034E-02  1.80298E-01 -3.76226E-02  2.26092E-02  1.74795E-02
NOTE BUSTER_TLS_L   tls1      1.45453E+00  1.61782E+00  8.45083E-01  9.10505E-02 -6.05438E-01  5.77972E-01
NOTE BUSTER_TLS_S   tls1      2.23750E-01 -2.44115E-01  3.38595E-01 -6.35630E-02  3.30303E-03 -2.70940E-01 -8.61559E-02 -8.56052E-02
NOTE BUSTER_TLS_O   tls1     -2.33111E+01 -8.37601E+01  1.13266E+01
NOTE BUSTER_TLS_FIX tls1 ALL
 
NOTE BUSTER_TLS_SET tls2 { B|* }
NOTE BUSTER_TLS_T   tls2     -1.96623E-01 -1.35074E-01  3.50236E-01  1.18743E-02  4.75488E-02 -1.48386E-02
NOTE BUSTER_TLS_L   tls2      1.48927E+00  9.09257E-01  1.09932E+00  7.78817E-01 -9.29773E-01 -7.15920E-01
NOTE BUSTER_TLS_S   tls2     -3.97061E-02  1.25383E-01 -1.13212E-01  1.50247E-01  1.99842E-01  3.65144E-02 -6.77258E-02  2.74050E-02
NOTE BUSTER_TLS_O   tls2      2.21121E+01 -7.20987E+01  3.46915E+00
NOTE BUSTER_TLS_FIX tls2 ALL

 For final position, Geometry function summary
     term                           number     weight       rms        GooF      function     fn/numb
     ================================================================================================
     BOND bond lengths (angs)       5676       2.000      0.0076       0.380      1642.85       0.289
     ANGL bond angles (degs)        7707       2.000        1.10       0.653      6577.11       0.853
     TORS split-harmonic tors       1281       0.000       17.15       1.075         0.00       0.000
     SINTOR sinusoidal tors         1281       2.000       17.15                  2232.49       1.743
     PLAN general plane (angs)       966       5.000      0.0175       0.874      3688.63       3.818
     BCORrelations (angs^2)         5676      10.000       0.891       0.318      5743.48       1.012
     CONT (bad contacts)               0       5.000                                 0.00
     IDEAL(ideal-dist contact)      5078       4.000                             10834.70
     CHIRAL (gelly semiharmon)       740       5.000                                57.27
     SIM similarity restraint for NCS and/or TARGET                              10578.06
     OCCSUM restraint on sum of occupanies                                           0.00       as none defined
     DISTAN utility restraints interatomic distance                                  0.00       as none defined
     UTILANGLE utility angle restraints                                              0.00       as none defined
     UTILTOR utility restraints on torsion angles                                    0.00       as none defined
     Peptide omega torsion angles                           3.27 degs - monitor only
 
 WARNING: Have |delta/sigma| deviations > 5.0 sigma. Number of outliers for each term: 
 WARNING:         3 bond angles.  Worst is    18.9 sigs    63.34 degs A|501:C10=C05=C04 (LIG)
 WARNING:         1 planes.       Worst is    12.4 sigs     0.25 Angs A|501:N03=C04=C05=C06=C07=C08=C09=C10 (LIG)
 WARNING:         1 idealD contacts. Worst     7.3 sigs     2.21 Angs A|501:C09=C11 (LIG)

 Weighted rms      0.0076         1.098  (temporary rms bond and angle for BUSTER to pick up!)
 Weighted intra-residual rms(bond) for StdProtein (excluding H)      0.0072
 Weighted intra-residual rms(bond) for Buffer     (excluding H)      0.0037
 Outputing geometry progress graph in plotmtv format to file:Geom_Res.mtv

 ############### Running final geometry/screen analysis (screen_final.txt)

 s/r gelly normal termination
```

Statistics and scale factors vs. cycle number
```

|  |  |  |
| --- | --- | --- |
|  | - Rxpct factors- <Log-Lik Gain>- GEOM Residuals- Ln Sigma_A_ | - Scale Factors- B Factors- Imperfection Factors |

```
   Final Geometry Information


---


### 2. Final results

Statistics and scale factors vs. cycle number
```

|  |  |  |
| --- | --- | --- |
|  | - Rxpct factors- <Log-Lik Gain>- GEOM Residuals- Ln Sigma_A_ | - Scale Factors- B Factors- Imperfection Factors |

```
   Average Figure of Merit
   Structure Factor Amplitudes


 # normal termination

  Program stopped.
```

closesok message: time spent in tree = 1882.95 (user) 39.22 (sys)

---

# Normal termination

```
 Resources usage summary:
     Time spent in client ...................  1882.96 (user)       39.25 (system)
     Time spent in scripts (    27 calls) ...     2.98 (user)        0.16 (system)
     Time spent in server ...................     0.00 (user)        0.01 (system)

     Max resident memory used (kb) .......... 469312
     Number of page faults .................. 0 (major)    1109147 (minor)
     Number of context switches ............. 3174 (niced)    6851 (other)
```
